# Gene Expression Profiling Reveals Enhanced Defense Responses in an Invasive Weed Compared to Its Native Congener During Pathogenesis

**DOI:** 10.3390/ijms20194916

**Published:** 2019-10-03

**Authors:** Bharani Manoharan, Shan-Shan Qi, Vignesh Dhandapani, Qi Chen, Susan Rutherford, Justin SH Wan, Sridharan Jegadeesan, Hong-Yu Yang, Qin Li, Jian Li, Zhi-Cong Dai, Dao-Lin Du

**Affiliations:** 1 Institute of Environment and Ecology, Academy of Environmental Health and Ecological Security, Jiangsu University, Zhenjiang 212013, China; biobharani@gmail.com (B.M.); qishanshan1986120@163.com (S.-S.Q.); lj060404002@126.com (J.L.); 2 School of the Environment and Safety Engineering, Jiangsu University, Zhenjiang 212013, China; chen1455526947@163.com (Q.C.); younghy524@163.com (H.-Y.Y.); liyunduo1024@163.com (Q.L.); 3 School of Biological, Earth and Environmental Sciences, University of New South Wales, Sydney, NSW 2052, Australia; wanjustinsh@gmail.com; 4 Environmental Genomics Group, School of Biosciences, University of Birmingham, Birmingham B15 2TT, UK; v.dhandapani@bham.ac.uk; 5 The Royal Botanic Gardens and Domain Trust, Mrs Macquaries Road, Sydney, NSW 2000, Australia; susan.rutherford@rbgsyd.nsw.gov.au; 6 The Robert H. Smith Institute of Plant Sciences and Genetics in Agriculture, The Robert H. Smith Faculty of Agriculture, Food and Environment, The Hebrew University of Jerusalem, Rehovot 761001, Israel; jsri.86@gmail.com; 7 Institute of Agricultural Engineering, Jiangsu University, Xuefu Road 301, Zhenjiang 212013, China.

**Keywords:** invasive weed, *Rhizoctonia solani*, gene expression, phytohormone signaling, systemic signal, plant defense

## Abstract

Invasive plants are a huge burden on the environment, and modify local ecosystems by affecting the indigenous biodiversity. Invasive plants are generally less affected by pathogens, although the underlying molecular mechanisms responsible for their enhanced resistance are unknown. We investigated expression profiles of three defense hormones (salicylic acid, jasmonic acid, and ethylene) and their associated genes in the invasive weed, *Alternanthera philoxeroides*, and its native congener, *A*. *sessilis*, after inoculation with *Rhizoctonia solani*. Pathogenicity tests showed significantly slower disease progression in *A*. *philoxeroides* compared to *A. sessilis*. Expression analyses revealed jasmonic acid (JA) and ethylene (ET) expressions were differentially regulated between *A*. *philoxeroides* and *A. sessilis*, with the former having prominent antagonistic cross-talk between salicylic acid (SA) and JA, and the latter showing weak or no cross-talk during disease development. We also found that JA levels decreased and SA levels increased during disease development in *A*. *philoxeroides*. Variations in hormonal gene expression between the invasive and native species (including interspecific differences in the strength of antagonistic cross-talk) were identified during *R. solani* pathogenesis. Thus, plant hormones and their cross-talk signaling may improve the resistance of invasive *A*. *philoxeroides* to pathogens, which has implications for other invasive species during the invasion process.

## 1. Introduction

Numerous plant species have been directly or indirectly introduced to new habitats as ornamentals, or as sources of food and fiber. However, many of these species have become invasive and pose a serious threat to agriculture, biodiversity and ecosystem function [1]. Following initial introduction, the spread of an invasive plant species could be further enhanced by global climate change (e.g., increasing CO_2_ emissions) [2,3]. Much of the previous research in invasion biology has focused on the ecological and evolutionary factors that contribute to plant invasions [4,5]. Due to the unavailability of genomic resources [6], the genetic factors underlying invasion success are still not well understood. 

Invasive plants may encounter novel abiotic and biotic stresses across the introduction–naturalization–invasion continuum. These stresses can affect their survival and reproduction, and can act as barriers to plant invasion. Successful invasive plant species possess various attributes (such as rapid adaptation, fast growth and spread, or high fecundity), and have effective defenses against natural enemies, which allow them to overcome barriers to invasion [7,8]. Higher resistance against generalist herbivores and pathogens may benefit invasive plants more than non-invasive species in new regions [8,9]. The enemy release hypothesis (ERH) and the biotic resistance hypothesis (BRH) have been proposed to explain the success and limitations that invasive species experience based on studies of novel plant–natural enemy interactions [10,11]. The ERH proposes that exotic plants will be less impacted by natural enemies compared to their native range because they have escaped their specialist herbivores or pathogens [12]. Therefore, the success of invasive plants in new environments can be attributed to the allocation of resources from defense to growth to outcompete other plants (evolution of increased competitive ability hypothesis or EICA) [13]. There has been a lot of support for the ERH, including reduced impacts by herbivores on invasive plants [14,15,16], as well as reduced attacks by above ground fungal and soil pathogens [14,15,17,18,19,20]. In contrast, the BRH states that native enemies should limit the growth of exotic plants in new ranges [21]. Many studies support the BRH [11,22,23,24,25], while some studies support both the ERH and BRH [26,27]. 

The improved performance of invasive plants in a competitive environment to enemy attack may be due to genetic changes acquired during the invasion process [6,28]. The use of genomic and transcriptomic technologies could identify the genetic architecture underlying the success of invasive plant species. A recent transcriptomic study was used to compare the gene expression profiles of introduced (North American) and native (European) populations of the Canada thistle, *Cirsium arvense*, in response to nutrient deficiency and shading [29]. This study identified significant differences in R-protein mediated defense and expression pattern between introduced and native populations of *C*. *arvense* [29]. Similarly, in the common ragweed (*Ambrosia artemisiifolia*), an invasive species to Europe, candidate genes were identified using oligonucleotide microarrays under light and nutrient stress conditions that were thought to contribute to invasiveness [30]. In addition, weedy sunflower genotypes of *Helianthus annuus* naturalized in the USA were tested for variations in gene expression compared to wild non-weedy species [31]. This study found extensive genetic differentiation between the two species [31]. However, until now, only a few investigations have been undertaken to elucidate the genomic mechanisms responsible for the adaptation of invasive plants to biotic stresses. 

Generally, a multitude of plant defense pathways are activated in response to microbial pathogens [32]. The first line of active defense occurs at the plant cell surface, when generalist microbe elicitors (the microbe or pathogen-associated molecular patterns, i.e., MAMP or PAMPs) are produced by pathogens such as, flagellin (from bacteria) [33], chitin (from fungal pathogens) [34], β-glucan (from oomycete pathogens) [35], or effectors (from specialized pathogens) [32]. Plants detect these elicitors by pattern-recognition receptors (PRRs) within the cell membrane, which leads to PAMP-triggered immunity (i.e., PTI) [36]. In the case of effectors, receptors with nucleotide-binding domains and leucine-rich repeats (NLRs or *R* genes) [37] are used, which lead to effector-triggered immunity (i.e., ETI) [38,39]. The evolutionary development of the plant immune system is represented as a Zig-zag model [40], where specialist pathogens often co-evolve with their host (see Han [41] for evolutionary dynamics between plants and pathogens). Invasive plants generally lack the need for defense against specialist pathogens in their new ranges (due to lack of co-evolution) [42] and therefore can invest more energy into growth or reproduction [12,13]. 

The timely recognition of the invading pathogen and a rapid, effective induction of defense responses are required for resistance to disease in plants [43]. Plant hormones play a key regulatory role in inducing defense responses shortly after the perception of a pathogen, through an extensive transcriptional reprogramming of genes involved in hormonal signaling [44]. In addition, the plant defense hormones play a critical role in response to adverse environmental conditions [45,46,47,48]. Abscisic acid (ABA) plays a central role in plant defense to abiotic stresses, such as salt and drought stress [49]. Salicylic acid (SA) is a major plant defense hormone induced by infections from biotrophic and hemi-biotrophic pathogens [50,51]. SA, jasmonic acid (JA), and ethylene (ET) also play key roles under biotic stresses [52]. These induced defenses and hormone signaling networks have been well characterized in model organisms and crop plants, such as *Arabidopsis* [53,54], tobacco [55] and tomato [56], but are not well known in invasive plants. For example, pyrosequencing was used to identify molecular signaling networks linked with paradormancy in underground vegetative buds of invasive *Cirsium arvense*. Interestingly, the plant hormone auxin and ABA-signaling was found to regulate paradormancy, allowing plants to resist weed control methods (e.g., chemical and biological controls), thereby enhancing their invasiveness [57]. Currently, there is a broad interest among invasion biologists to unravel the genetic mechanisms of resistance and defense responses of invasive plants. In this study, we hypothesize that invasive plant species have higher or enhanced resistance to microbial pathogens compared to native congeners. We also predict that the endogenous defense mechanism and signaling (especially the defense hormones SA, JA, and ET) play a crucial role in resistance, thereby benefiting the invasion success of invasive plants.

The alligator weed, *Alternanthera philoxeroides* (Martius) Grisebach, is an amphibious stoloniferous perennial herb [58]. It is native to South America [59] and was first introduced to China in the late 1930s as a forage crop from Japan [60]. It is the most noxious invasive plant in China [61,62] where it is a significant weed in rice farms, causing an estimated agricultural loss of $75 million per year [63]. Populations of *A. philoxeroides* growing in China have extremely low genetic diversity, which is attributed to the predominance of a single genotype (likely due to a single recent introduction) and extensive vegetative propagation by cuttings since being introduced [60,64].

*Alternanthera philoxeroides* is highly vulnerable to insect herbivore attack. For example, more than 15 generalist insects were found to feed on *A. philoxeroides* in China [11]. A specialist beetle, *Agasicles hygrophila* (alligator weed flea beetle) from South America was introduced to the USA [59] and China [11] to control *A. philoxeroides*. However, only a few disease incidences in *A. philoxeroides* have been reported. For example, species of *Nimbya* have been found to cause leaf and stem spot on *A. philoxeroides* in Australia [65]. *Nimbya alternantherae* has been identified as a biocontrol agent in Brazil [66], while species of *Fusarium* have been used as a biocontrol for alligator weed in China [67]. *Rhizoctonia solani* has been shown to be pathogenic to *A. philoxeroides*, and has also been found to infect a related species, *Alternanthera sessilis*, in the USA [68] and India [69]. Earlier preliminary pathogenicity screening tests showed that *R. solani* (ACCC 30374) is more virulent on *A. philoxeroides* compared to *Fusarium oxysporum* f.sp *cubense* (ACCC 36369) (SS Qi, unpublished data). Therefore, *A. philoxeroides* represents a good model to study pathogen resistance and defenses in an invasive plant species following invasion. 

In this study, we aimed to isolate the defense hormones (SA, JA, and ET) and associated genes in invasive *A. philoxeroides* and its native congener *A. sessilis* to test for differences in gene expression between the species against a generalist necrotrophic fungus, *Rhizoctonia solani*. Although signaling of these defense hormones and their cross-talk in response to pathogens are well documented in *Arabidopsis* and other model plant species [53,54,55,56], our study is the first to examine these phenomena in wild populations of a co-occurring invasive and native congener pair. Furthermore, divergence in gene expression between the species may allow us to identify patterns of defense signaling that may be responsible for enhanced resistance in invasive *A. philoxeroides*. Specifically, we ask the following questions: (1) Is invasive *A. philoxeroides* less susceptible to the pathogen (*R. solani*) compared to its native congener *A. sessilis*? (2) Are there differences in gene expression between the native and invasive species after inoculation with *R. solani*? (3) Are there differences in resistance between infected and un-infected neighboring leaves? To address these questions, we performed in vitro and in planta leaf inoculations using *R. solani*. Six hormones and their responsive genes (three JA, two SA, and one ET) were successfully isolated from both invasive *A. philoxeroides* and native *A. sessilis* for expression analysis using RT-qPCR in response to three treatments. The first included *R. solani* inoculation for susceptibility tests, the second comprised un-infected samples for systemic resistance tests, and the third included hormone pretreatments using SA, JA, and ET (prior to inoculation with *R. solani*).

## 2. Results

### 2.1. Disease Development in A. philoxeroides Was Delayed Compared with A. sessilis

**Figure 1:**
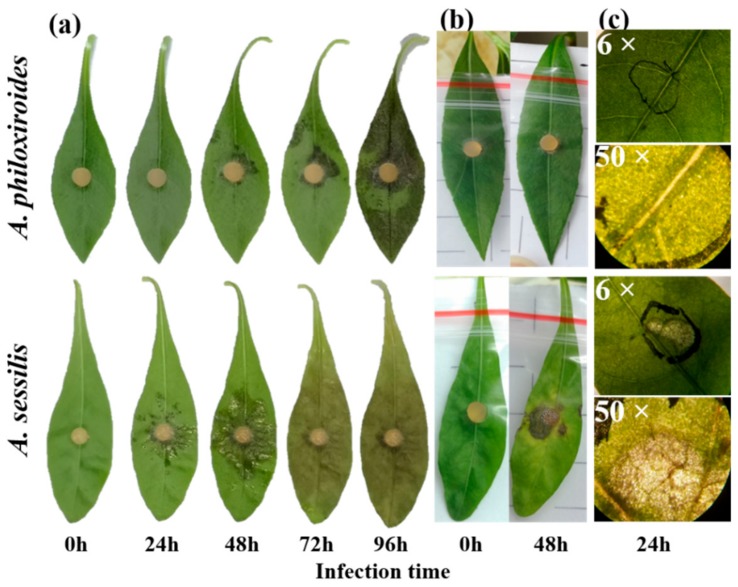
Disease symptoms caused by *R. solani* on invasive *A. philoxiroides* and native *A. sessilis* showing (**a**) in vitro detached leaf assay; (**b**) in planta pathogenicity test; and (**c**) microscopic observation of the infected area on leaves (at 24 hpi). Four-week-old plant leaves were inoculated with mycelium plugs of *R. solani* and disease symptoms were observed at various time intervals (24, 48, 72 and 96 hpi). For in planta tests, plastic bags were placed on the leaves after inoculation to avoid drying of plugs and moisture development for better infections. In (**c**), the inoculated area is circled with a black marker. After removing plugs, threads of fungal mycelium can be seen on the leaf surface in *A. philoxiroides*, indicating fungal colonization. This is in contrast to the clear necrotic disease lesions in *A. sessilis*.

The pathogenicity of *R. solani* was tested on invasive *A. philoxiroides* and native *A. sessilis*. Disease symptoms and necrotic lesions developed on leaves of both plant species (Figure 1a). However, *A. philoxiroides* showed slower disease progression compared to *A. sessilis* (Figure 1a,b). *Alternanthera sessilis* exhibited prominent disease symptoms at 24 hpi, whereas *A. philoxiroides* had no visible symptoms and mycelium was only observed at the site of inoculation under the microscope, indicating minimal fungal colonization (Figure 1c). Necrotic lesions were noticeable only at 48 hpi in *A. philoxiroides* (Figure 1a).

**Figure 2:**
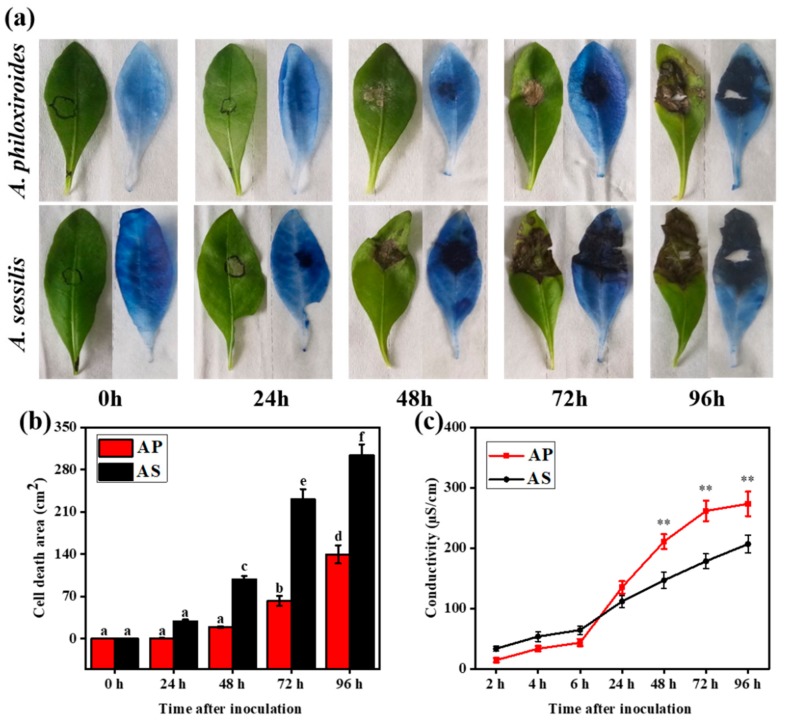
Trypan blue staining and ion leakage tests of *R. solani* infected leaves from invasive *A. philoxiroides* (AP) and native *A. sessilis* (AS) at different time intervals after inoculation. (**a**) Before and after staining with trypan blue for visualizing dead plant leave tissues. Circled areas on leaves are at 0 h (un-inoculated control) and 24 h (inoculated) before staining. (**b**) Cell death areas represented by means ± standard errors (SE) from three biological replicates with different letters representing the groups that were significantly different from other groups as determined by a one-way analysis of variance (ANOVA), followed by a multiple comparison using Duncan’s method (*p* < 0.05). (**c**) Electrolyte leakage of leaf discs infected by *R. solani* from 2 h to 96 h measured using an electrolytes’ conductivity meter. Electrolytic conductivity increased in native AS during earlier time intervals; however, it increased in invasive AP at later time intervals compared to the un-inoculated control (0 h). Error bars indicate means ± SE (number of disks = 12 for each species) and two discs from each plant representing a total of six biological replicates for each species. Asterisks indicate significantly different from native plants using Duncan’s method (*p* < 0.01).

Infected leaves stained with trypan blue clearly highlighted the region of cell death on the leaves (Figure 2a). *Alternanthera philoxiroides* had a significantly lower cell death area compared to *A. sessilis* across all time intervals post-inoculation with *R. solani* (Figure 2b). Furthermore, the amount of ion leakage was significantly higher at earlier time periods (<24 hpi) in *A. sessilis* compared to *A. philoxiroides*, which showed higher leakages at a later time interval (>48 hpi; Figure 2c). Overall, these results suggest that, although both plants are susceptible to *R. solani*, the invasive *A. philoxiroides* showed slower disease progression compared to the native *A. sessilis*.

### 2.2. Expression Divergence of Defense Hormone Genes between A. philoxiroides and A. sessilis

**Figure 3:**
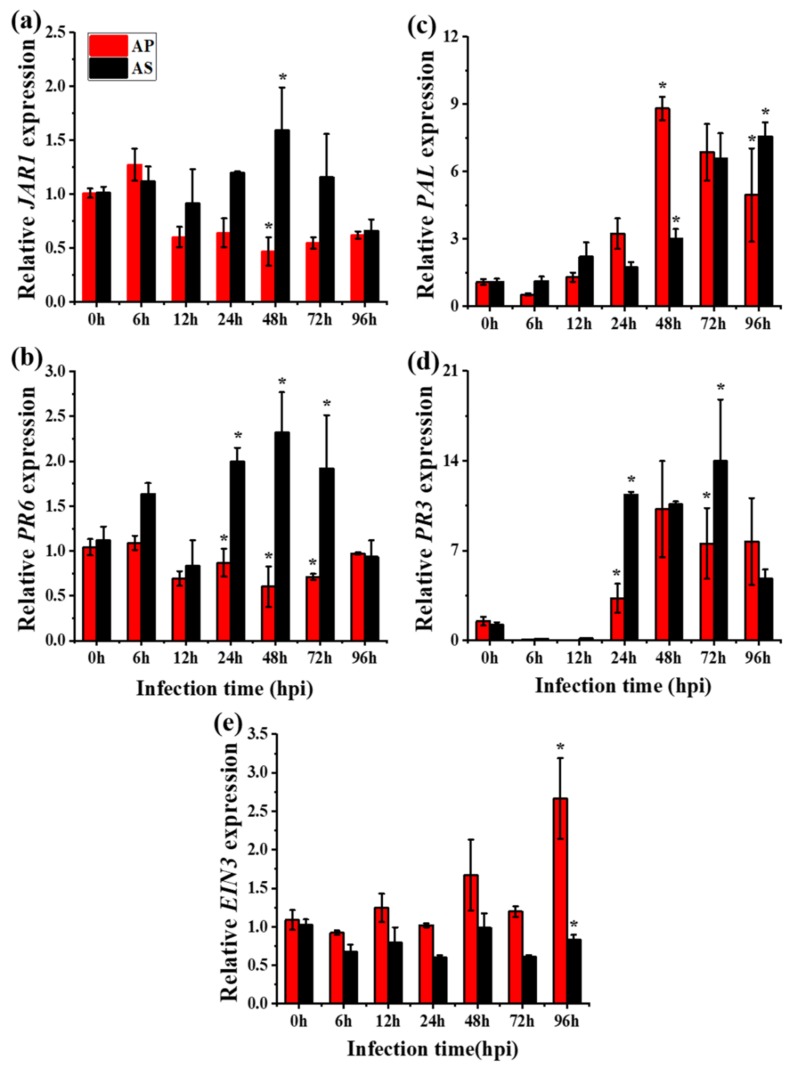
qPCR analysis of salicylic acid (SA), jasmonic acid (JA) and ethylene (ET)- responsive gene expressions between invasive *A. philoxiroides* (AP) and native *A. sessilis* (AS). Four-week-old plants were infected with *R. solani* and the samples were harvested for RNA extractions at the indicated time intervals (0 to 96 hours) after inoculations. Un-inoculated leaves were used as a control (0 h). Specific primers were used for *JAR1* (**a**), *PR6* (**b**), *PAL* (**c**), *PR3* (**d**) and *EIN3* (**e**) with *Actin* (control) as shown in Supplementary Table S2. Values represent means ± SE from three biological replicates. The asterisks (*) represent significantly different levels at each time period and were determined using one-way ANOVA, followed by a multiple comparison using Duncan’s method (*p* < 0.05).

**Figure 4:**
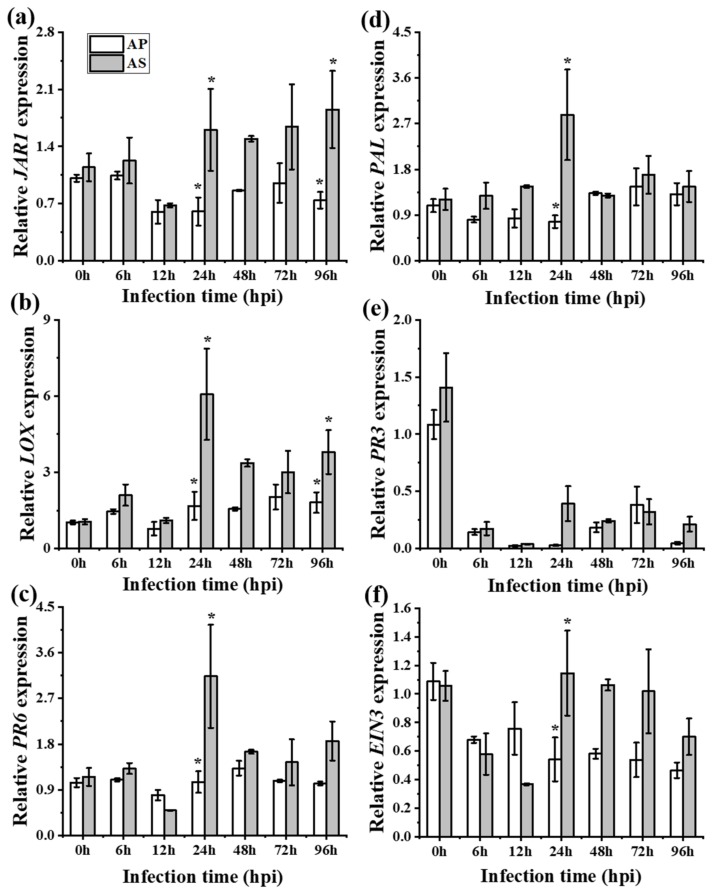
Expression analysis of SA, JA and ET-responsive genes for systemic acquired resistance tests between *Alternanthera philoxiroides* (AP) and *A*. *sessilis* (AS). Four-week-old plants were infected with *R. solani* and samples of healthy un-inoculated leaves were sampled for RNA extractions at the indicated time intervals (0 to 96 hours). 0 h is from the samples of plant completely un-infected for control. Relative expression of *JAR1* (**a**), *LOX* (**b**), *PR6* (**c**), *PAL* (**d**), *PR3* (**e**), *EIN3* (**f**) and *Actin* (control) were tested using gene specific primers at the indicated time intervals. Error bars show ± SE from three biological replicates and the asterisks (*) represent significantly different levels, which were determined via a one-way ANOVA, followed by a multiple comparison using Duncan’s method (*p* < 0.05).

**Figure 5:**
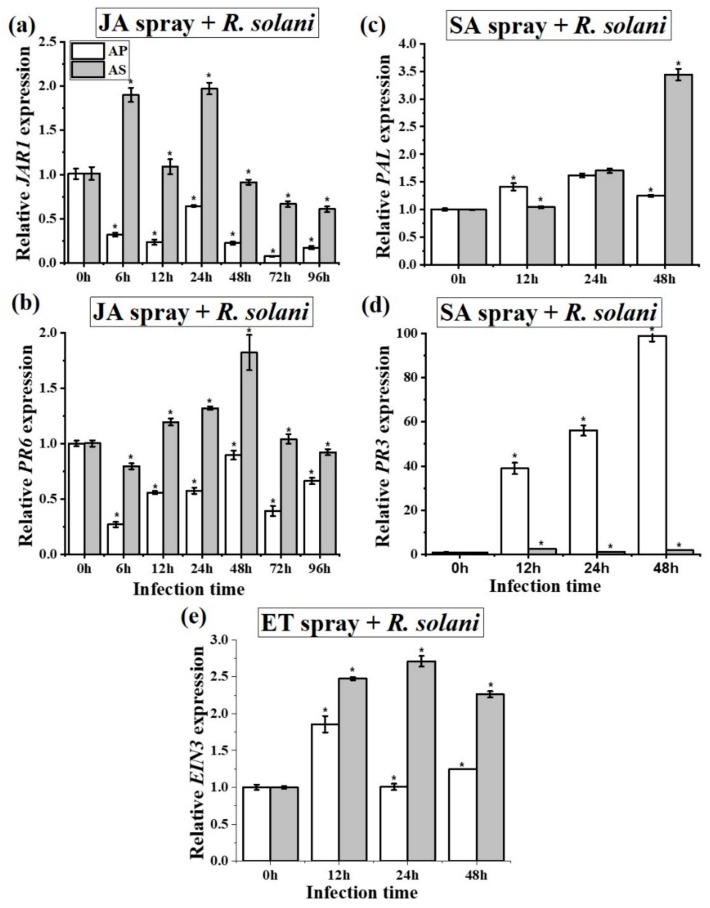
Expression analysis of SA, JA and ET-responsive genes for hormone pre-treatment tests between *Alternanthera philoxiroides* (AP) and *A*. *sessilis* (AS). Four-week-old plants were sprayed with MeJA pretreatment (**a**,**b**) SA pretreatment (**c**,**d**), and ET pretreatment (**e**) before being inoculated with *R. solani*. Samples were harvested for RNA extractions at the indicated time intervals after inoculations. Un-inoculated leaves were used as a control (0 h). Specific primers were used for *JAR1* (**a**), *PR6* (**b**), *PAL* (**c**), *PR3* (**d**) and *EIN3* (**e**) with *Actin* (control) being tested using gene specific primers at the indicated time intervals. Values represent means ± SE from three biological replicates. Asterisk (*) represent significantly different levels at each time period and was determined via a one-way ANOVA, followed by a multiple comparison using Duncan’s method (*p* < 0.05).

Expressions of all six genes (*PAL*, *PR3*, *LOX*, *JAR1*, *PR6,* and *EIN3*) were quantified in both the local and systemic leaves of the two species against *R. solani* (the fold–change ratio of defense hormones and their responsive genes are presented in Table S1). Expression differences were observed in both infected local (Figure 3) and systemic leaves (Figure 4), as well as the hormone content (Figure S1) between *A. philoxiroides* and *A. sessilis* during disease development caused by *R. solani* (Table 1 and Table S1). Furthermore, expression differences were also observed in the hormone pre-treatment group between the two species (Figure 5). The key difference was that JA and ET-signaling was differentially regulated in *A. sessilis*. JA was partially suppressed in *A. sessilis*, whereas there was a consistent reduction in expression in *A. philoxiroides* (Figure 3a,b). ET-*EIN3* expression was reduced in *A. sessilis* but was induced in *A. philoxiroides* (Figure 3e). SA level was induced in both species (Figure 3c,d). In addition, weaker or no antagonistic cross-talk was observed between SA (Figure 3c,d) and JA-signaling (Figure 3a,b) in *A. sessilis*. In contrast, there was strong cross-talk in *A. philoxiroides* (Figure 3 and Table 1).

### 2.3. Functional Analysis of Defense Hormone Gene Sequences between A. philoxiroides and A. sessilis

Six hormones and responsive gene sequences of both invasive and native plants from our study (Table S3) were searched in the NCBI nucleotide database and their identity was checked against other plant species in the Amaranthaceae (Table S4). The output of each gene sequence was obtained to predict a high confidence protein coding sequence from six reading frames (Table S4). Furthermore, a gene from each hormone (*PAL*, *JAR1*, and *EIN3*) was selected on the basis of core signaling component for detailed functional analysis in both *A. philoxiroides* and *A. sessilis*. Each sequence was assembled to a single long contig to predict protein coding genes along with mRNA and the amino acid translation (Table S5). We aimed to compare each gene sequence with other closely related species in Amaranthaceae, and also to compare between *A. philoxiroides* and *A. sessilis* (Figures S2–S5). A comparative phylogenetic analysis of the three hormone genes revealed high conservation within Amaranthaceae (including *Beta vulgaris* and *Spinacia oleracea*, Figure S2). We predicted the conserved domains and motifs in each gene sequence and analyzed how conserved they were across species (see Figure S3 for motifs and Figure S4 for domains). We found that multiple sequence alignments of each amino acid sequence showed high levels of gene conservation between *A. philoxiroides* and *A. sessilis*, and high conservation between the two study species and other related species (Figure S5). 

### 2.4. R. solani Suppresses Jasmonic Acid Signaling for Disease Promotion in A. philoxiroides

**Figure 6:**
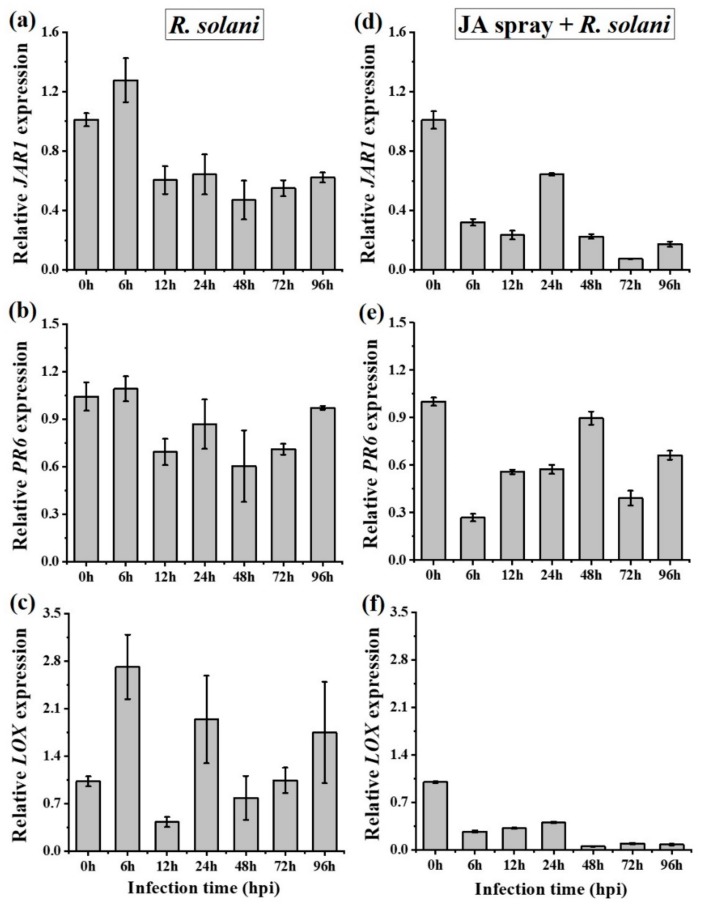
qPCR analysis of JA-dependent gene expression in *A. philoxiroides*. Four-week-old plants were infected with *R. solani* (**a**–**c**), or sprayed with MeJA pretreatment before being inoculated with *R. solani* (**d**–**f**). Samples were harvested for RNA extractions at the indicated time points (0 to 96 hours). 0 h is the un-inoculated control. Relative expression of JA responsive genes: *JAR1* (**a** and **d**), *PR6* (**b** and **e**), *LOX* (**c**,**f**) were tested with specific primers for *A. philoxiroides* as described in the Methods and Supplementary Table S2. Values represent means ± SE from three biological replicates.

To examine the role of JA-signaling, expression levels of three JA-dependent transcripts, *LOX* (Lipoxygenase), *JAR1* (JA amido synthetase 1), and *PR6* (Proteinase inhibitor), were tested in *A. philoxiroides* following inoculation with *R. solani*. We found the expressions of *JAR1* and *PR6* were reduced in *A. philoxiroides*, whereas *LOX* expression was inconsistent at each time after inoculation (Figure 6a–c). At 6 hpi, all three (*LOX*, *JAR1,* and *PR6*) levels had increased (Figure 6a–c; Table S1). Plants subjected to the MeJA hormone pretreatment also showed reduced expressions across all three JA genes at all time intervals (Figure 6d–f). Overall, our results suggest that JA signaling may be responsible for resistance to *R. solani* in *A. philoxiroides* because JA was induced earlier (up to 6 hpi). During disease progression (after 48 hpi), *R. solani* may overcome JA-resistance signaling by suppressing the defensive responses in *A. philoxiroides* (Figure 6).

### 2.5. Salicylic Acid and Ethylene Signaling Enhances Disease Susceptibility in A. philoxiroides

**Figure 7:**
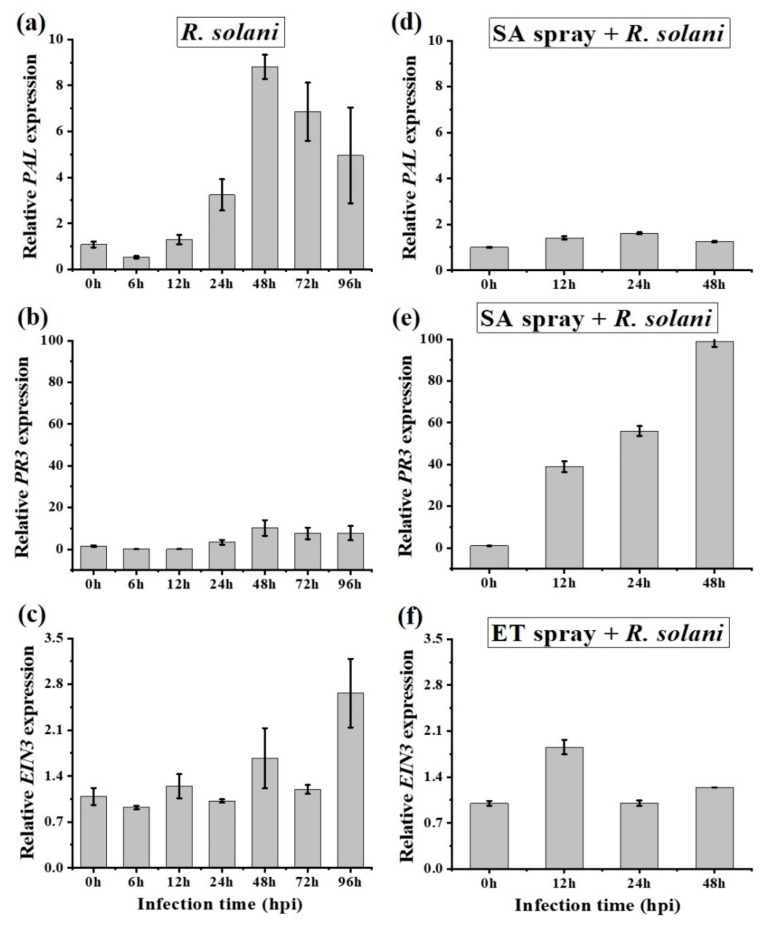
qPCR analysis of SA and ET-responsive gene expression in *A. philoxiroides*. Four-week-old plants were infected with *R. solani* (**a**–**c**), or were sprayed prior to *R. solani* inoculations with SA pretreatment (**d**,**e**) or ET pretreatment (**f**). Samples were harvested for RNA extractions at the indicated time points after the inoculations. 0 h is the un-inoculated control. qPCR was performed with specific primers for SA-*PAL* (**a**,**d**), *PR3* (**b**,**e**), ET-*EIN3* (**c**,**f**) and *Actin* (control) as described in the Methods and shown in Supplementary Table S2. Values represent means ±SE from three biological replicates.

To determine the role of SA in plant defense response, we tested expression levels of two SA transcripts in *A. philoxiroides*: *PAL* and *PR3* (Supplementary Table S3). We found that the expression of both transcripts consistently increased at each time interval, with the exception of 6 hpi (where expression decreased, Figure 7a,b). The reduced expression at 6 hpi was in contrast to the positive induction of JA transcripts (Figure 6a–c). After inoculation, both *PAL* and *PR3* were also induced in SA pretreated plants (Figure 7d, e). However, *PR3* showed very high levels of expression (up to 100-fold at 48 hpi, Figure 7e and Table S1), suggesting that SA promotes disease development in *A. philoxiroides* following inoculation with *R. solani*.

The ET transcript, *EIN3*, displayed a minor increase in expression over time in both the *R. solani* infected and ET-pretreatment samples (Figure 7c,f). Specifically, the expression of *EIN3* was reduced at 6 hpi (similar to SA, Figure 7a). Overall, the results suggest ET-signaling may act synergistically with SA, thereby promoting disease susceptibility in *A. philoxiroides*.

### 2.6. Signaling Cross-Talk between SA, JA and ET in A. philoxiroides during Interactions with R. solani

**Figure 8:**
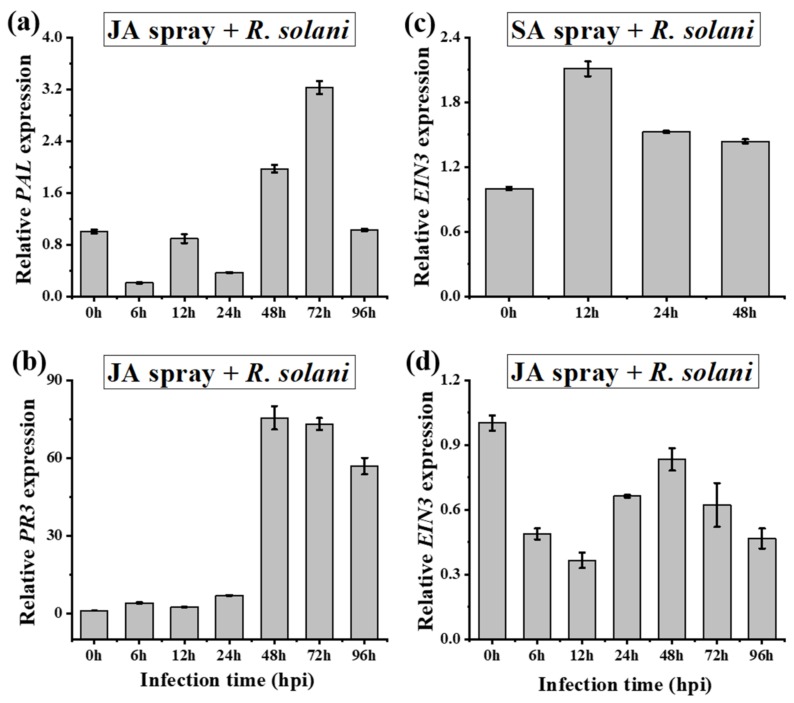
Signaling cross-talk analysis between SA, JA and ET-responsive gene expressions during hormone pretreatments, before *R. solani* inoculations in *A. philoxiroides*. Four-week-old plants were sprayed with MeJA (**a**, **b** and **d**) and SA (**c**). Samples were harvested for RNA extractions at the indicated time points. Un-inoculated leaves were used as a control (0 h). qRT-PCR was performed with specific primers for SA-*PAL* (**a**), *PR3* (**b**), ET-*EIN3* (**c**,**d**) and *Actin* (control) as described in the Methods and shown in Supplementary Table S2. Values represent means ± SE from three biological replicates.

We investigated the cross-talk between hormone signaling pathways in response to *R. solani* infection and hormone pretreatments. We observed that JA (*LOX, JAR1*, and *PR6*) and SA (*PAL* and *PR3*) transcripts had prominent antagonistic cross-talk in *A. philoxiroides* at both earlier (at 6 hpi) and later (>24 hpi) time intervals following *R. solani* inoculations (Figure 6a–c and Figure 7a,b). In addition, MeJA pretreated *A. philoxiroides* showed decreased expressions in all three JA transcripts (Figure 6d–f), and also displayed higher expression levels of SA transcripts (*PAL* and *PR3*) (Figure 8a,b and Table S1). These results indicate a clear antagonistic cross-talk between SA and JA pathways in *A. philoxiroides* after inoculation with *R. solani*.

As described above for ET-*EIN3*, gene expression was induced in both treatments in *A. philoxiroides* (Figure 4c, f). In addition, we also tested the expression of *EIN3* with other hormone pretreatments in *A. philoxiroides*. For example, SA pretreated plants induced *EIN3* expression similar to the SA-*PAL* and *PR3* transcripts (Figure 8c). In contrast, MeJA pretreated plants showed a decrease in *EIN3* expression, similar to the JA (*LOX*, *JAR1*, and *PR6*) transcripts (Figure 8d). The differential expression of *EIN3* to SA and MeJA suggests that ET may be regulated by both hormones depending on the type of infection and treatment. Other combinations of hormone signaling and their cross-talk gene expressions are presented in Supplementary Table S1. 

To further test whether *R. solani* influences the hormones (SA, JA and ET) during pathogenesis in *A. philoxiroides*, we quantified the endogenous contents of each hormone in *R. solani* infected plants at each time interval (0, 6, 12, 24, 48, 72 and 96 hpi) using ELISA. We detected a 6.9-fold higher SA content (Figure S6a) and a 2.2-fold higher JA content (Supplementary Figure S6b) at 96 hpi compared to the control un-inoculated samples. ET was at a moderate level (about 3.2-fold higher) compared to both SA and JA in the infected plants (Supplementary Figure S6c). The levels of each hormone increased, as time since infection increased (Supplementary Figure S6).

### 2.7. R. solani Induced Resistance Trade-Offs between SA and JA-Signaling in A. philoxiroides

**Figure 9:**
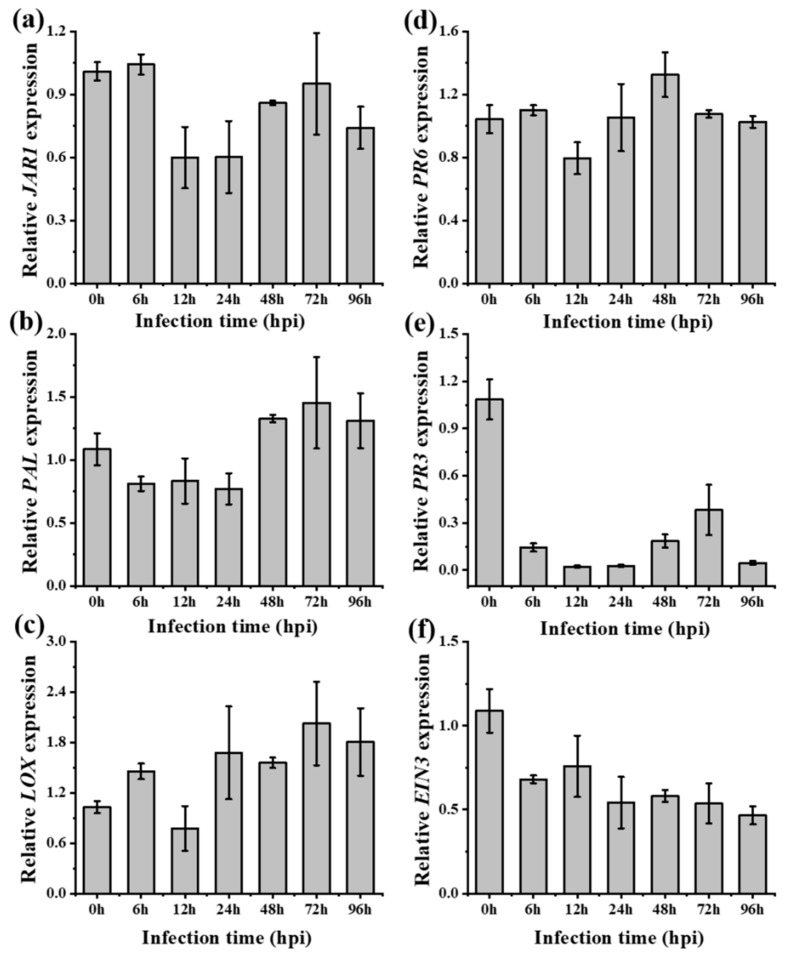
Expression analysis of SA, JA and ET-responsive genes for systemic acquired resistance tests in *A*. *philoxiroides*. Four-week-old *A. philoxiroides* were infected with *R. solani* and samples of healthy un-inoculated leaves were collected for RNA extractions (at time intervals from 0 to 96 hours). 0 h is from the samples of plant completely un-infected for control. Relative expression of *JAR1* (**a**), *PAL* (**b**), *LOX* (**c**), *PR6* (**d**), *PR3* (**e**) and *EIN3* (**f**) with *Actin* (control) genes were tested using specific primers at the indicated time intervals. Values represent means ± SE from three biological replicates.

To identify and correlate whether hormonal cross-talk provides signals to adjacent leaves from local infected tissues, we examined the resistance trade-offs between SA and JA-signaling in *A. philoxiroides*. Our findings suggest that SA was induced during *R. solani* pathogenesis, which initiated antagonistic cross-talk to JA at the local infected leaves in *A. philoxiroides* (Figure 6 and Figure 7). Investigating neighboring leaves for this cross-talk may provide information regarding how trade-off signals are regulated during infection in invasive *A. philoxiroides*. Therefore, we quantified the expression levels of all six genes in neighboring un-inoculated leaves across all time intervals. We found that JA-*JAR1* showed reduced expressions at each time interval (Figure 9a). In contrast, increased expression of SA-*PAL* from 48 hpi was detected (Figure 9b). The cross-talk between these transcripts in the neighboring un-inoculated leaf was similar to the infected leaves. However, other transcripts of JA-*LOX* and *PR6* were induced in the same plants (Figure 9c,d), while SA-*PR3* was much reduced (Figure 9e). These findings suggest that the key signaling component of SA (*PAL*) and JA (*JAR1*) engage in antagonistic cross-talk during pathogenesis. The ET-*EIN3* expression was reduced at each time interval (Figure 9f).

## 3. Discussion

Plants are exposed to numerous biotic and abiotic stresses in their environment. Invasive plants may have evolved to adapt to these stresses and to fluctuating environmental conditions via plasticity in growth and development [6,28,70]. Studies investigating the molecular mechanisms responsible for invasion success are scarce in non-model plant species, but can now be investigated due to the availability of genomic technologies. In our study, we isolated partial gene sequences of key defense hormones in two non-model plant species (Table S6). These novel template gene sequences allowed us to investigate hormone signaling and their cross-talk within and between species to better understand the resistance of an invasive species to a globally distributed necrotrophic fungal pathogen. There were three major findings in our study: (1) *R. solani* successfully infected the invasive *A. philoxeroides* and its native congener *A. sessilis,* with disease development being much slower in *A. philoxeroides* compared to *A. sessilis*; (2) there were interspecific differences in hormone gene expression (including hormone signaling and cross-talk) following inoculation by *R. solani*; and (3) there were differences in the hormone signaling and their cross-talk between infected local and the un-infected systemic leaves.

### 3.1. Differential Expression between Invasive A. philoxiroides and Native A. sessilis during Disease Development

**Figure 10:**
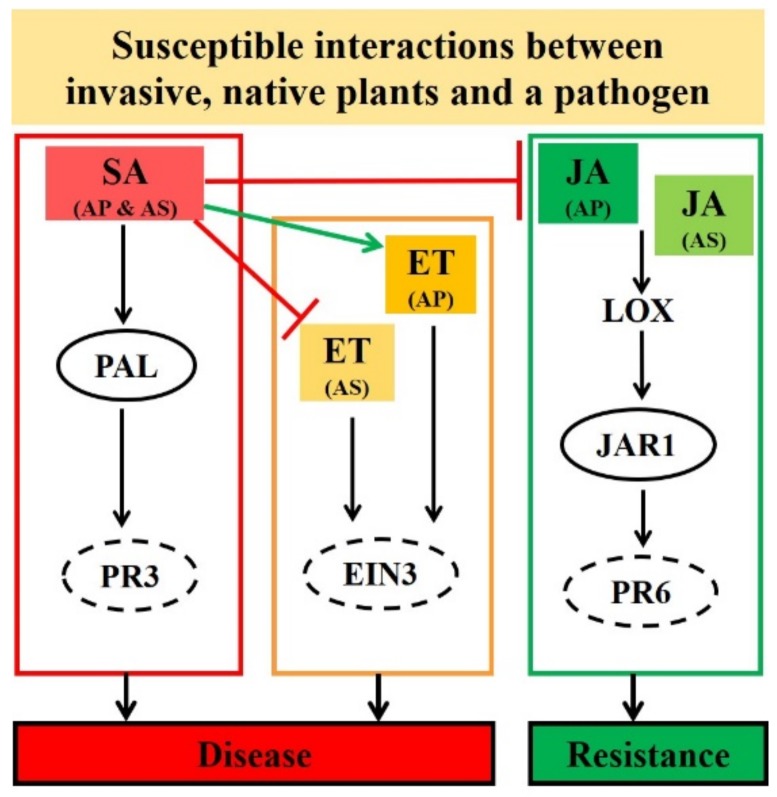
Proposed model showing response of defense hormone genes, salicylic acid (SA), jasmonic acid (JA), and ethylene (ET) during pathogenesis in an invasive *Alternanthera philoxeroides* (AP) and native *A. sessilis* (AS). We investigated the molecular interactions between *R. solani* compared to two species (focusing on defense hormone signaling). Two key differences in hormone gene expressions were identified between species during pathogenesis. Firstly, the JA and ET-signaling was differentially regulated between species (partially suppressed in *A. sessilis*, whereas there was a consistent reduction in expression in *A. philoxiroides*, shown as difference in height and color of JA). Reduction in JA-dependent *LOX*, *JAR1* and *PR6* expressions during disease development suggested that JA-signaling is responsible for resistance to *R. solani*. Secondly, ET-*EIN3* expression was reduced in *A. sessilis*, but was induced in *A. philoxiroides* (shown as difference in height and color of ET). SA level was induced in both species. The elevated levels of SA in both species during disease development suggest that the unknown virulence factor from pathogenic *R. solani* may potentially target SA. This in turn may affect the antagonistic effect between SA and JA/ET differentially between species (shown as a different arrow between SA and ET). Circled SA-*PAL* and JA-*JAR1* show antagonistic effects in both the local inoculated and neighboring systemic leaves. Dotted circles (*PR3*, *EIN3* and *PR6*) represent differential regulation in both inoculated and un-inoculated systemic sites.

We found that there were interspecific differences in JA and ET-signaling, as well as in the degree of antagonistic cross-talk between SA and JA (which was stronger in the invasive species and weaker in the native species). This variation in the expression of defense hormone genes between species suggests *R. solani* may affect host plant defense hormones, specifically by inducing the major defense hormone SA (which may regulate other hormones, such as JA and ET, differently between plants for successful infection of plant tissue, Table 1). Our findings also suggest that the infection of *R. solani* was enhanced in the native *A. sessilis*, perhaps due to the weaker antagonistic cross-talk between SA and JA (which may allow faster or enhanced disease development to occur in the native species). Furthermore, our results indicate that the stronger antagonistic cross-talk between SA and JA in the invasive *A. philoxiroides* may delay disease progression. To our knowledge, this is the first study that demonstrates a necrotrophic pathogen influencing plant defense hormone pathways in an invasive clonal weed differentially compared to a native congener (Figure 10). A previous study [30] identified several genes that may be involved in the introduction success of invasive *A. artemisiifolia*. Of the 180 genes identified in this earlier study, some genes were found to be involved in the metabolism of plant hormone signaling and biosynthesis (e.g., lipoxygenases and cytokinins-zeatin o-glucosyltransferase) [30]. Gene expression differences between the invasive and native species suggested that invasive species may have evolved to stressful conditions during the invasion process [29,30,31]. 

The differences in defense hormone signaling between the invasive and native species observed in our study during pathogenesis may be the result of interspecific genetic variation. It would be interesting to study how differences in genetic variation between the invasive *A. philoxeroides* (which has previously been found to have low genetic diversity in China due to predominantly clonal vegetative propagation, [60,64]) and native *A. sessilis* (which may have higher genetic diversity in its native range, although this needs to be confirmed) may influence the resistance response of each species to pathogens. The data from our study provide an appropriate baseline for investigating this line of inquiry in the future. 

### 3.2. Changes in Defense Hormone Gene Expression during Pathogenesis in A. philoxeroides 

Our study demonstrates how a common and widespread pathogen regulates plant defense hormones (SA, JA, and ET) allowing for the successful infection of an invasive species, *A. philoxeroides* (using RT-qPCR). Our results showed that both JA-*JAR1* and *PR6* transcript expressions decreased during disease development following inoculation with *R. solani* AG4 HGI (Figure 6a,b), indicating that JA-signaling pathway is likely to be involved in plant resistance to the pathogen. There is much evidence to suggest that endogenous JA is triggered against necrotrophic pathogens [71,72]. For example, gene expression changes induced by *R. solani* AG1 IA in resistant and susceptible rice plants have been reported and it has also been shown that JA plays an important role in disease resistance [73]. In the present study, we found that the JA biosynthetic *LOX* gene was inconsistent in expression (Figure 6c). This was consistent with the findings of a previous study [74], where it was found that *LOX* expression increased approximately 6-fold in response to virulent and avirulent strains of *Pseudomonas syringae*.

In our study, *R. solani* induced SA (*PAL* and *PR3*), which may antagonize JA during pathogenesis in invasive *A. philoxeroides* (Figure 6a,b and Figure 7a,b). Earlier studies have shown cross-talk between hormone signaling pathways can greatly help the plant to regulate defense responses to a wide range of pathogens [75,76,77]. The predominant cross-talk observed between SA and JA is antagonistic [50,52]. For example, an exogenous application of SA was found to inhibit JA-induced proteinase inhibitor expression in tomato [54,78], whereas MeJA treatment inhibited SA-induced acidic *PR* gene expression in tobacco [55]. Like in our study, previous studies have found that phytopathogens can take advantage of the cross-talk between SA and JA allowing them to successfully infect plants [79,80]. For example, the hemi-biotrophic pathogen *P. syringae* was shown to manipulate antagonism between SA and JA by producing coronatine, which is a phytotoxin that mimics plant JA to suppress SA. This mechanism promotes disease infection in *Arabidopsis* and tomato [81]. The necrotrophic *Botrytis cinerea* produces exopolysaccharide (EPS), a virulence factor that elicits SA to activate antagonism to JA, thereby enhancing the ability of the fungus to infect tomato [56]. Our results are consistent with the findings of El Oirdi et al. [56], although the virulence factor from *R. solani* needs to be investigated further. A follow-up study by El Oirdi et al. [56] showed SA-signaling can also contribute to disease development (caused by another necrotrophic pathogen, *Alternaria solani*) in tomato [82]. It has also been found that the SA pathway might contribute to disease development (i.e., a *SAR8.2k* gene induced by SA may be involved in disease susceptibility caused by *R. solani* in tobacco [83]). 

*PR* genes are often associated with specific signaling pathways and their expression can be regulated by different plant hormones [84]. In our study, at 48 hpi, the SA-inducible *PR3* showed a 10-fold higher expression to *R. solani* infection (Figure 7b), a 100-fold higher expression for SA pretreatment (Figure 7e), and a 75-fold higher expression for JA pretreatment (Figure 8b). This increase in SA-inducible *PR3* supports the hypothesis that SA enhances disease development by suppressing JA in *A. philoxeroides*. 

Our results indicate that, as with SA, *EIN3* (an active form of the ET transcription factor) [77] enhances disease development in *A. philoxeroides* (Figure 7c). Although *EIN3* was induced at moderate levels, its expression was consistently induced over time during inoculation (Figure 7c,f). Previous studies have shown that ethylene is a potential modulator of plant pathogen defenses [85]. JA and ET together form an effective defense against necrotrophs [86], and can act positively or negatively with SA, depending on the specific pathogen interactions [87]. For example, ET modulates the antagonism between SA and JA pathways, and is mediated by NPR1 [53]. ET induces SA-responsive *PR1* gene expression in *Arabidopsis* [88]. In contrast, ET (*EIN3* and EIL1) represses the SA biosynthesis gene *ICS/SID2*, thereby reducing SA accumulation [89].

### 3.3. Hormonal Cross-Talk and Systemic Resistance in A. philoxeroides during R. solani Pathogenesis

Invasive plants may encounter multiple pathogens with different infection strategies in their non-native range. Hormonal cross-talk provides signals to adjacent leaves from local infected tissues to resist a forthcoming infection [90,91,92]. This systemic defense is effective against pathogens with a similar attacking strategy [93]. For pathogens with a different infection mode, hormonal cross-talk between pathways (specifically SA and JA) plays a critical role in plant resistance [90,94]. The biotrophic pathogen inducing SA can activate antagonism to JA in infected local and systemic tissues, which in turn favors insect herbivores or necrotrophic pathogens [91].

In our study, trade-offs between the SA and JA pathways was clearly observed in infected local leaves (Figure 6 and Figure 7). This trade-off was lower in neighboring leaves (Figure 9). Only the core signaling component of SA-*PAL* and JA-*JAR1* showed antagonism in the neighboring leaves, whereas other gene transcriptions (i.e., JA-*LOX* and *PR6*) were not suppressed (Figure 9). Our results are consistent with the finding of Spoel et al. [90], who reported that *P. syringae* suppressed JA-mediated resistance to *Alternaria brassicicola* at the infected site. However, this antagonism was at modest levels in neighboring tissues in *Arabidopisis* [90]. It was suggested that, while antagonism between SA and JA was moderate, their cross-talk expression was detected in systemic tissues [90]. However, spatial separation (local, adjacent or systemic), time (immediate or delayed), pathogen type (biotroph or necrotroph), and inoculation dosage are important factors determining systemic resistance trade-offs in plants [91].

## 4. Materials and Methods

### 4.1. Plant Species and Pathogens

*Alternanthera philoxeroides* (Amaranthaceae) is one of the 100 worst invasive species in the world [59]. Climate modeling suggests that many regions around the world are suitable habitat for the growth of *A. philoxeroides*, including areas in Southeast Asia, Southern Africa, and Southern Europe [95]. In China, it has successfully invaded 19 provinces since its introduction (usually on roadsides and lakeshores) [96].

*Alternanthera sessilis* (L.) DC. is native to China, and was used for comparison with *A. philoxeroides* in our study [22]. Ramets of *A. philoxeroides* and *A. sessilis* were randomly collected in August (summer) 2016 from naturally occurring sites in Fuzhou National Forest Park (119°17′12′′E, 26°9′35′′N, Figure S7) and propagated in a greenhouse at Jiangsu University, Zhenjiang, China. Healthy stems with two nodes and without roots were planted in 250 g of sterilized sand in plastic pots (8 × 7 × 5 cm). Fixed volumes (30 mL) of distilled water or full-strength Hoagland liquid nutrient solution [97] were supplemented alternatively every two days to maintain optimal growth conditions. 

*Rhizoctonia solani* is a ubiquitous soil-borne necrotroph that causes significant yield loss in many economically important crops globally [98]. The most common symptom is the ‘damping-off’ of seedlings or failure of infected seeds to germinate. The *R. solani* AG4-HGI (accession ACCC 30374) used in this study was obtained from the Agriculture Culture Collection of China (Agricultural and Microbial Culture Collection Management Center, Beijing, China). This strain was further sequenced to identify the specific anastomosis group (AG4-HGI), which was confirmed by PCR amplification of AG common and subgroup specific primers [99], and internal transcribed spacer (ITS) primers [100]. The fungus was grown and sub-cultured on potato dextrose agar (PDA) and incubated for five days at 28 °C.

### 4.2. Isolation of Putative Hormone-Responsive Genes for RT-qPCR

Partial gene sequences of six hormones and responsive genes (one for ET, two for SA and three for JA) were successfully isolated from both *A. philoxiroides* and *A. sessilis*. Although there is currently a lack of hormone specific sequence information for many invasive and native plant species (including *Alternanthera* species), the species from our study are in the Amaranthaceae family (which was used for sequence isolation). Twenty-one hormones and their responsive gene sequences (i.e., eight for SA, seven for JA, and six for ET) were retrieved from species in the Amaranthaceae (Table S6). Each gene sequence from species in the Amaranthaceae was aligned using Clustal Omega [101] for primer design in the conserved region (using Primer3) [102] (for initial screening). We also performed a phylogenetic analysis of these sequences (Figure S2) to compare with other species.

Plant leaf material was ground using liquid nitrogen, and 50–100 mg of ground tissue was used for DNA extraction using the Rapid Plant Genomic DNA Isolation Kit (Sangon Biotech, Shanghai, China). Genomic DNA was further precipitated by adding sodium acetate and ethanol and purified using the method of Dellaporta et al. [103]. PCR was performed, and DNA was purified using the UNIQ-10 Column MicroDNA Gel Extraction Kit (Sangon Biotech, Shanghai, China) prior to sequencing. A total of six genes (i.e., *PAL* and *PR3* for SA; *LOX*, *JAR1* and *PR6* for JA; *EIN3* for ET) were successfully isolated based on the PCR amplification of similar amplicon lengths obtained from both *A. philoxiroides* and *A. sessilis* (Table S3). Furthermore, the coding (exonic) region of each isolated gene sequence was confirmed by cDNA PCR. We performed a NCBI nucleotide search of each of the isolated sequences and found that they showed closest homology to sequences of species in the Amaranthaceae. RT-qPCR primers (amplicon length < 200 bp) were designed using web-based Integrated DNA Technologies (IDT)-Primer Quest [104] from initially screened sequences of each gene. Primers with similar amplification efficiency in the cDNA of both invasive and native plants were used in RT-qPCR. All the primer sequences used for RT-qPCR are listed in Supplementary Table S2.

### 4.3. Plant Inoculations with R. solani for RT-qPCR

Pathogenicity tests consisted of detached (in vitro) and in planta leaves from four-week old stem cuttings of *A. philoxeroides* and *A. sessilis* inoculated with 5-mm agar plugs (*R. solani* culture). Both detached and in planta leaf assays were performed using the method used by El Oirdi and Bouarab [105]. Inoculated leaves of in planta inoculations with mycelium plugs were covered with clear zip-lock plastic bags to maintain high humidity. Detached and in planta disease symptoms were photographed every day, up to five days after inoculation.

In planta inoculations of *A. philoxeroides* and *A. sessilis* were used for gene expression experiments by quantitative RT-qPCR. Inoculated leaf samples were harvested at different time intervals, including 0, 6, 12, 24, 48, 72, and 96 h post-inoculation (hpi). There were three biological replicates per time interval (i.e., one plant per replicate each for *A. philoxeroides* and *A. sessilis*). The 0 hpi time interval refers to un-inoculated control samples. All inoculated plants were grown at 25 °C and 70% humidity with a 16 h photoperiod. Harvested leaf material at different time intervals was quickly frozen in liquid nitrogen for RNA extractions. For the systemic acquired resistance (SAR) tests, samples of un-inoculated neighboring leaves (i.e., younger leaves just above the inoculated leaf) were investigated. For SAR gene expression experiments, samples of three biological replicates were taken per time interval (0, 6, 12, 24, 48, 72, and 96 hpi). That is, one plant per replicate for each species was investigated. The experiments of *R. solani* infection and SAR were repeated twice.

The effect of each hormone (SA, JA, and ET) on resistance to *R. solani* was tested using RT-qPCR by spraying each hormone two days before inoculation with *R. solani*. Hormone pretreatments were performed on four-week old plants of *A. philoxeroides* and *A. sessilis*. There were three treatments in this experiment group for each species (SA, JA, and ET pretreatments). Hormones SA (0.5 mM, BBI Life Sciences, Shanghai, China), methyl jasmonate (MeJA, 0.1 mM, Sigma-Aldrich, St. Louis, MO, USA), and ethephon (ET, 0.5 mM, BBI Life Sciences, Shanghai, China) were dissolved in water. Hormones were sprayed directly on plant leaves (in each treatment group) until drenched (surface run-off) once per day, for three consecutive days. All treatment plants were grown at 25 °C and 70% humidity with a 16 h photoperiod until they were harvested. Each of the hormone pretreated samples was harvested at different time intervals: 0, 6, 12, 24, 48, 72, and 96 hpi with *R. solani*. The hormone pre-treatment samples for SA and ET spray were unable to be collected after 72 and 96 h. This was because the ET-spray samples wilted early and could not be sampled (i.e., healthy leaves could not be obtained). The control treatment (0 hpi) consisted of only water pretreated and un-inoculated. There were three biological replicates for each time interval (i.e., one plant per replicate) for each species. All harvested samples were quickly frozen in liquid nitrogen for RNA extractions. The experiment was repeated twice.

### 4.4. RT-qPCR Analysis

Total RNA was isolated from leaves of invasive and native plants in each treatment using the TaKaRa MiniBEST Plant RNA Extraction Kit according to the manufacturer’s instructions (Takara, Shiga, Japan). First-strand cDNA was synthesized from 500 µg total RNA using PrimeScript RT Master Mix (Takara, Japan). The targets were amplified using primers that are listed in Table S2. RT-qPCR was performed using SYBR Premix Ex-Taq (Takara, Japan) on CFX96 Real-Time PCR Detection System (Bio-rad). Melt-curve analysis was performed after each PCR run to ensure specific amplification of each gene-specific primer. To identify the suitable housekeeping gene, we tested three reference genes, *β-tub* (β-tubulin), *EF1α* (Elongation factor 1-alpha), and *Act* (Actin), in *R. solani* infected samples based on Cycle threshold (Ct) difference and the coefficient of variance method [106]. Primers are listed in Table S2. *Actin* had the lowest Ct difference in *R. solani* infected samples across different time intervals (Manoharan B. et al., unpublished data) and was used for normalizing the expression level of each target gene. Relative gene expression was calculated using the comparative 2^−ΔΔ*CT*^ method [107]. Three biological replicates were used for each gene and each reaction was independently replicated three times.

### 4.5. Hormone Content Quantification

The endogenous hormone contents (SA, JA, and ET) was determined for *R. solani* infected *A. philoxiroides* and *A. sessilis*. Fungal inoculations were performed on four-week old plants (as described above). Samples collected at different time intervals (0, 6, 12, 24, 48, 72, and 96 hpi) were subjected to hormone quantification by Lengton Bio. Tec. Co., Ltd. (Shanghai, China) using the ELISA method [97]. The 0 hpi time interval refers to the un-inoculated control samples.

### 4.6. Quantification of Plant Cell Death by Trypan Blue Staining and Ion Leakage Assay

To visualize dead plant cells and to measure the cell death area of infected leaves, trypan blue staining was performed on the leaves of four-week old infected *A. philoxiroides* and *A. sessilis*. Leaves were removed at each time interval (0, 24, 48, 72, and 96 hpi) and stained with lactophenol-trypan blue, followed by de-staining with saturated chloral hydrate using the method of Koch and Slusarenko [108]. Samples were photographed every day before and after staining to measure the diameter of the infected area using ImageJ [109]. To further confirm cell death, ion leakage assay was performed on infected leaves of both species using an electrolytic conductivity meter (model P772) following the procedure outlined in Hatsugai and Katagiri [110]. 

### 4.7. Flanking Sequence Isolation and Bioinformatic Analyses

Six isolated DNA sequences from both *A. philoxeroides* and *A. sessilis* (Table S3) were searched in the NCBI non-redundant protein database using BLAST+ (version 2.2.31) to compare with other plant species. BLASTx output of each sequence was submitted to OrfPredictor [111] to identify the best matching open reading frame (ORF) sequence [110] (Table S4). Furthermore, one gene from each of the hormones, *PAL* (SA), *JAR1* (JA), and *EIN3* (ET) was selected for additional flanking sequence isolation for both *A. philoxiroides* and *A. sessilis* (for detailed function and bioinformation analysis). Flanking (5′ and 3′) sequences were isolated from a known sequence region (i.e., initially isolated sequences, Table S2) using 5′ Genome walking and 3′ RACE (Rapid Amplification of cDNA Ends) techniques, following the methods outlined in the respective kit instructions (Genome Walking Kit, code 6108 and 3′-Full RACE Core Set with PrimeScript RTase, code 6106, Takara, Shiga, Japan). Isolated sequences from both techniques were assembled for each gene with the corresponding initial sequence using CAP3 sequence assembly program with the following parameters: base quality cut-off for clipping value of 12, overlap length cut-off value ≥20, overlap percent identity ≥75, and overlap similarity score ≥500 [112]. The assembled single long contig was selected for annotating protein coding gene with ab-initio method and *Beta vulgaris* gene-specific parameters using the FGENESH online tool [113]. Predicted CDS and peptide sequences were reconfirmed by aligning to the NCBI RefSeq nucleotide and protein database using mega BLAST and BLASTp search tools. Clustal-W from MEGA 7.0.26 was used for multiple sequence alignment of each peptide sequence with other related species. A maximum likelihood phylogenetic tree was constructed with 1000 bootstrap replicates (Figure S2) [114]. In addition, motifs were searched for each peptide sequences using MEME suite [115] with the default options. The conserved domain was searched using the NCBI database with an expected value of 0.010000 [116]. 

### 4.8. Data Availability

The partial gene sequences isolated in this study were deposited in the GenBank under accession IDs: MK790145 to MK790156. The *Rhizoctonia solani* was sequenced to identify the specific anastomosis group and was deposited under GenBank accession ID: MK801228.

## 5. Conclusions

During the invasion process, an invasive species from a small founding population may face difficulties due to adverse abiotic and biotic stresses, impacting its survival and reproduction. Invasive species can undergo genetic changes to overcome these natural barriers. Our findings are consistent with previous studies, which identified that many stress induced genes are differentially expressed between invasive and native plant species [29,30,31]. Our study advances our current understanding of hormone resistance to a widespread pathogen in an invasive species compared to its native congener. In addition, our findings provide insights into the significance of invasive plant defense pathway genes that may have evolved during the invasion process. The manipulation of host defense hormones in favor of pathogen colonization in native plant species may be a mechanism where invasive species gain an advantage over co-occurring native congeners, and this should be the focus of future research. Further studies are required to identify the currently unknown virulence factors (i.e., pathogen effectors or toxins that mimic plant hormone coronatine from *R. solani* and other pathogens) that may affect the signaling pathways differentially between invasive and native species [56]. More specifically, research is needed on resistance (*R*) genes, such as the non-expresser of PR genes 1 (NPR1) of hormone SA, which is a major transcriptional activator that activates antagonism to JA or ET during disease development [117]. 

## Figures and Tables

**Table 1:** Expression differences in defense hormones and their responsive genes in the invasive *A. philoxeroides* and native *A. sessilis* during *R. solani* pathogenesis. See Table S1 for fold–change ratios of each gene for each treatment in both the invasive and native species.

Treatment	*Alternanthera philoxeroides*	*Alternanthera sessilis*
*Rhizoctonia solani* (Infected local leaves)	- JA was suppressed (*JAR1* (Figure 3a) and *PR6* (Figure 3b))	- JA was partially suppressed (*JAR1* (Figure 3a) and *PR6* (Figure 3b))
	- SA was induced (*PAL* (Figure 3c) and *PR3* (Figure 3d))	- SA was induced (*PAL* (Figure 3c) and *PR3* (Figure 3d))
	- ET was induced (*EIN3* (Figure 3e))	- ET was suppressed (*EIN3* (Figure 3e))
	- SA may promote disease development by suppressing JA and by inducing ET (Figure 3) - Strong antagonistic cross-talk between SA and JA - Stronger antagonistic resistance cross-talk between SA and JA may be reason for the delayed disease development (Figure 1 and Figure 2)	- SA may promote disease development but by partially suppressing JA and by completely suppressing ET (Figure 3) - No strict or strong antagonistic cross-talk between SA and JA - Lack of stronger antagonistic resistance effect between SA and JA may be reason for the enhanced disease development (Figure 1 and Figure 2)
*Rhizoctonia solani* (Systemic leaves)	- JA-*JAR1* was suppressed (Figure 4a) - JA-*LOX* (Figure 4b) and *PR6* (Figure 4c) was induced but lower than *A. sessilis*	- JA was induced (*JAR1* (Figure 4a), *LOX* (Figure 4b) and *PR6* (Figure 4c))
	- SA-*PAL* was induced from 48 hpi (Figure 4d) - SA-*PR3* was suppressed at all time (Figure 4e)	- SA-*PAL* was induced at all the indicated time (Figure 4d) - SA-*PR3* was completely suppressed (Figure 4e)
	- ET-*EIN3* was suppressed all the time (Figure 4f)	- ET-*EIN3* was partially suppressed (Figure 4f)
	- An antagonism between SA-*PAL* and JA-*JAR1* was observed (Figure 4)	- No antagonism between SA-*PAL* and JA-*JAR1* was observed (Figure 4)
*Rhizoctonia solani* (Hormone contents)	- Endogenous contents of SA, JA and ET were higher. For example, at 96 hpi, SA, JA and ET contents were significantly higher than control samples (Figure S1)	- Endogenous contents of SA, JA and ET were comparatively lower. For example, at 96 hpi, SA, JA and ET contents were significantly higher than control samples (Figure S1)

## Supplementary Materials

All supplementary material from this study is available online at https://www.mdpi.com/1422-0067/20/19/4916/s1, including Figure S1: Endogenous hormone salicylic acid (SA), jasmonic acid (JA), and ethylene (ET) contents in both *Alternanthera philoxiroides* and *A. sessilis* after *Rhizoctonia solani* inoculations. Figure S2: Comparative phylogenetic analysis of the selected hormone genes in both the invasive and native species, as well as other closely related species. Figure S3: Identification of conserved motifs in selected genes from both invasive and native species. Figure S4: Identification and alignment of the conserved domain in each of the selected genes from both invasive and native species. Figure S5: Multiple sequence alignments of predicted amino acid sequences of both *Alternanthera philoxeroides* and *A. sessilis* along with other closely related plants. Figure S6: Endogenous hormone Salicylic acid (SA), jasmonic acid (JA), and ethylene (ET) contents in *Alternanthera philoxiroides* after infected with *Rhizoctonia solani*. Figure S7: Sampling site of invasive *Alternanthera philoxeroides* and native *A*. *sessilis*. Table S1: Fold-change ratio of defense hormones and their responsive genes under different treatment conditions in invasive *Alternanthera philoxeroides* compared to native *A. sessilis*. Table S2: Primers used for quantitative RT-qPCR. Table S3: List of putative defense hormones and responsive genes isolated in both *Alternanthera philoxeroides* and *A. sessilis*. Table S4: Sequence information of six defense hormones and responsive genes isolated from *Alternanthera philoxeroides* and *A. sessilis*. Table S5: Functional analysis of three hormone genes in both *Alternanthera philoxeroides* and *A. sessilis*. Table S6: Screening and isolation of defense hormones and responsive gene sequences from both invasive *Alternanthera philoxeroides* and native *A*. *sessilis*.
