# Supplementary material for: Gene Expression Profiling Reveals Enhanced Defense Responses in an Invasive Weed Compared to Its Native Congener During Pathogenesis
Source: Int J Mol Sci. 2019 Oct 3;20(19):4916. doi: 10.3390/ijms20194916 (PMC6801458; doi:10.3390/ijms20194916)
Supplement: Supplementary file 1 [file ijms-20-04916-s001.pdf]

International Journal of *Molecular Sciences*

MDPI

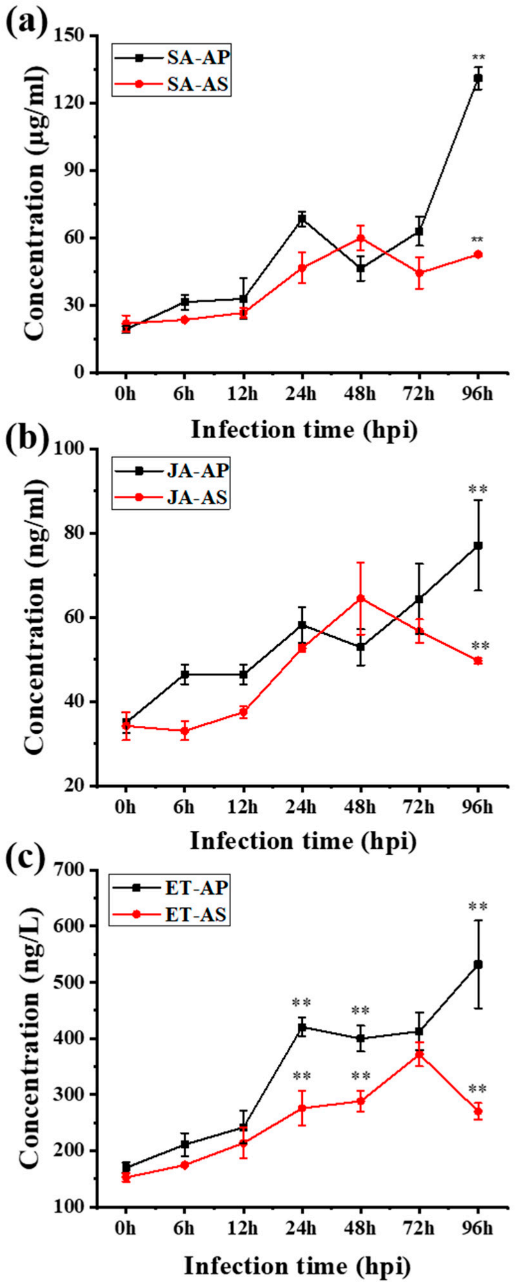

Figure S1. Endogenous hormone Salicylic acid (SA), jasmonic acid (JA), and ethylene (ET) contents in both *Alternanthera philoxiroides* and *A. sessilis* after *Rhizoctonia solani* inoculations. (a) SA, (b) JA and (c) ET. Four-week-old plants were inoculated with *R. solani* and un-inoculated leaves were used as a control (0h). Samples were harvested at the indicated time points for quantification using ELISA. Values are means ± SE (n = 4). The different letters indicate significant difference in hormone levels compared with control un-inoculated plant leaves assessed by Duncan's test at $P < 0.01$. Abbreviations: AP - *A. philoxeroides*, AS - *A. sessilis*.

(a) *PAL* (SA)

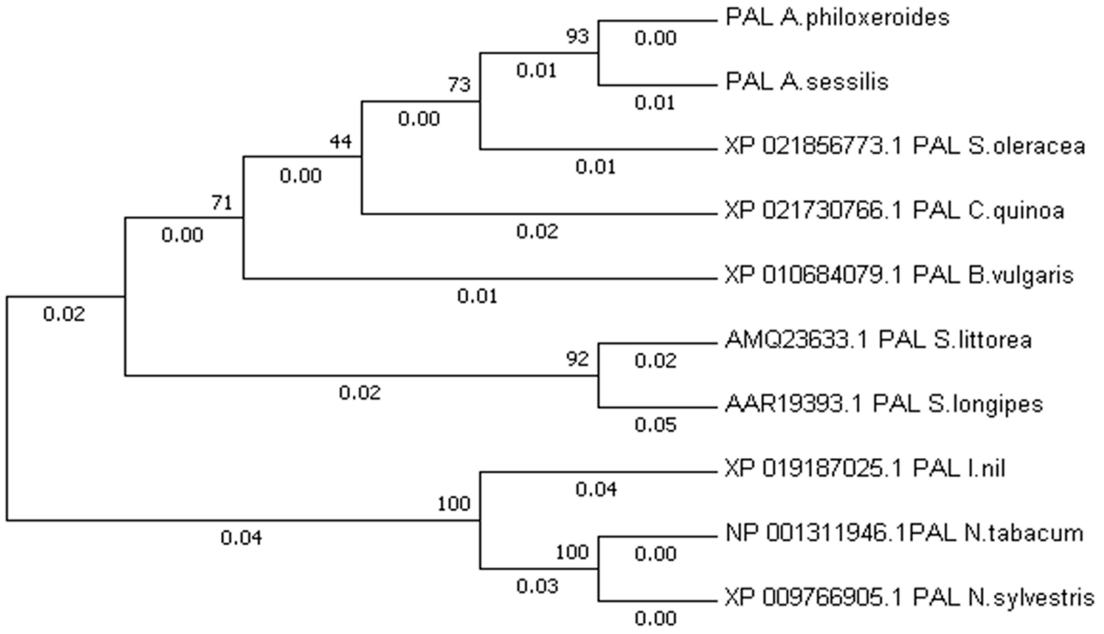

(b) *JAR1* (JA)

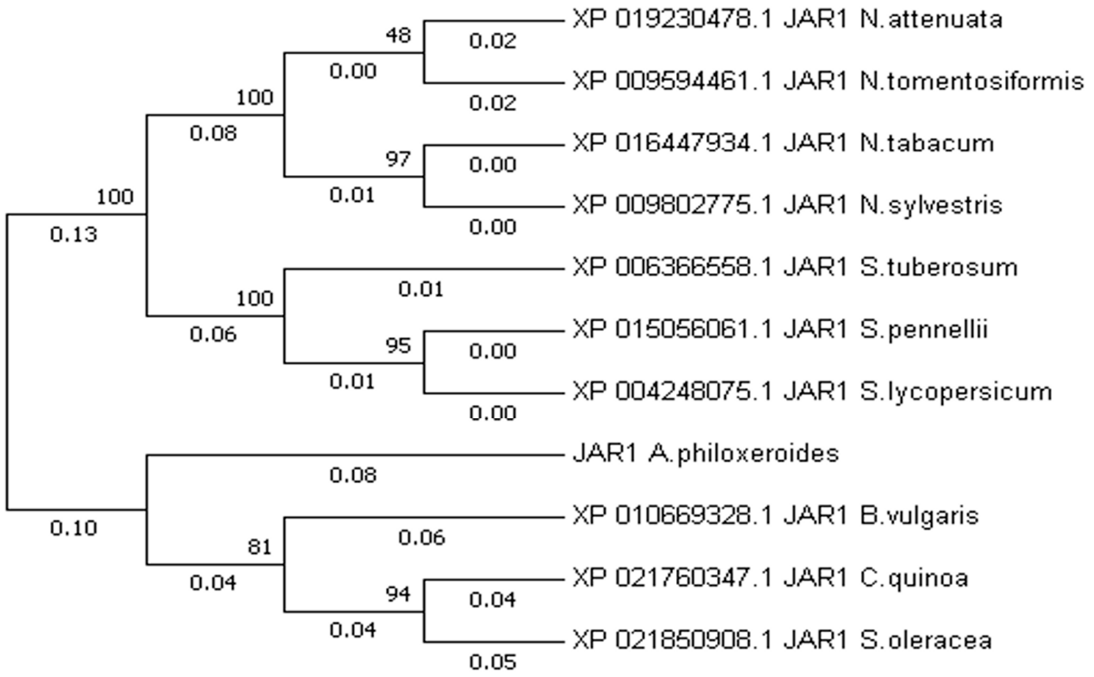

(c) *EIN3* (ET)

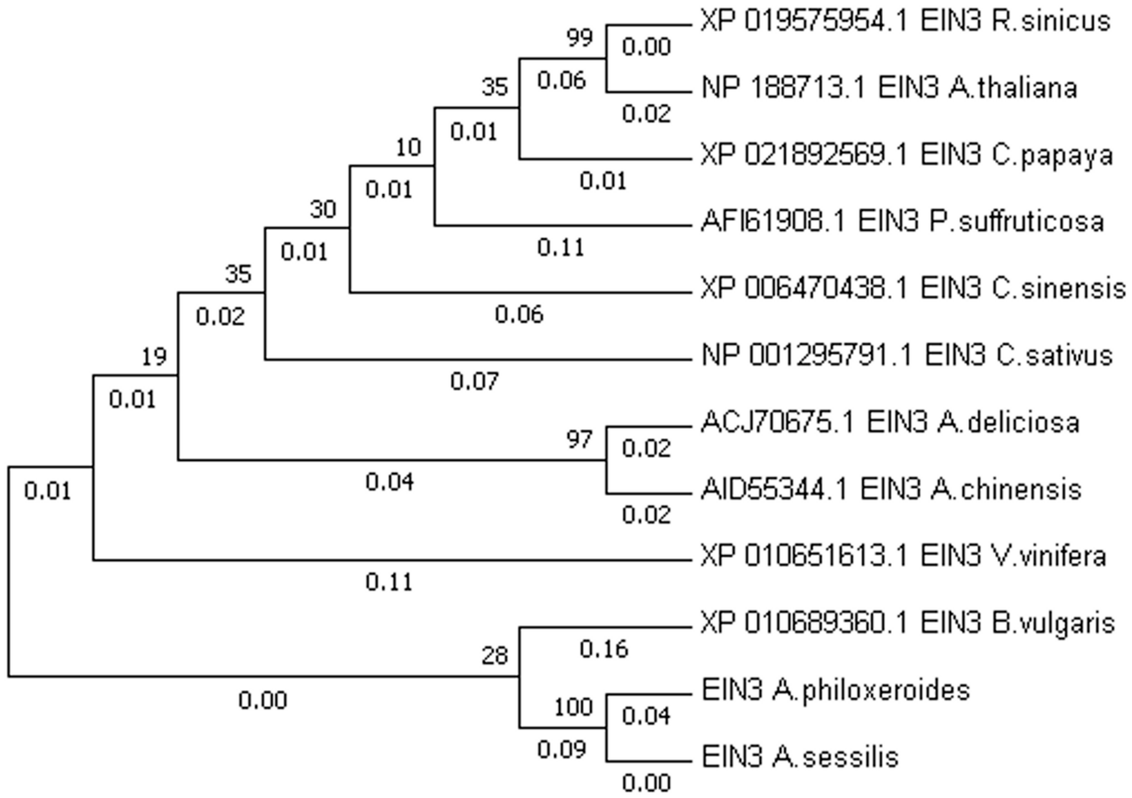

Figure S2. Comparative phylogenetic analysis of the selected hormone genes in both the invasive and native species, as well as other closely related species. A maximum likelihood analysis was performed for each gene: (a) *PAL* (Salicylic Acid), (b) *JAR1* (Jasmonic Acid), (c) *EIN3* (Ethylene). A maximum likelihood phylogenetic tree was constructed with 1000 bootstrap replicates. Bootstraps are shown on the nodes of the trees. Genetic distances are shown below each branch.

(a) *PAL* (SA)

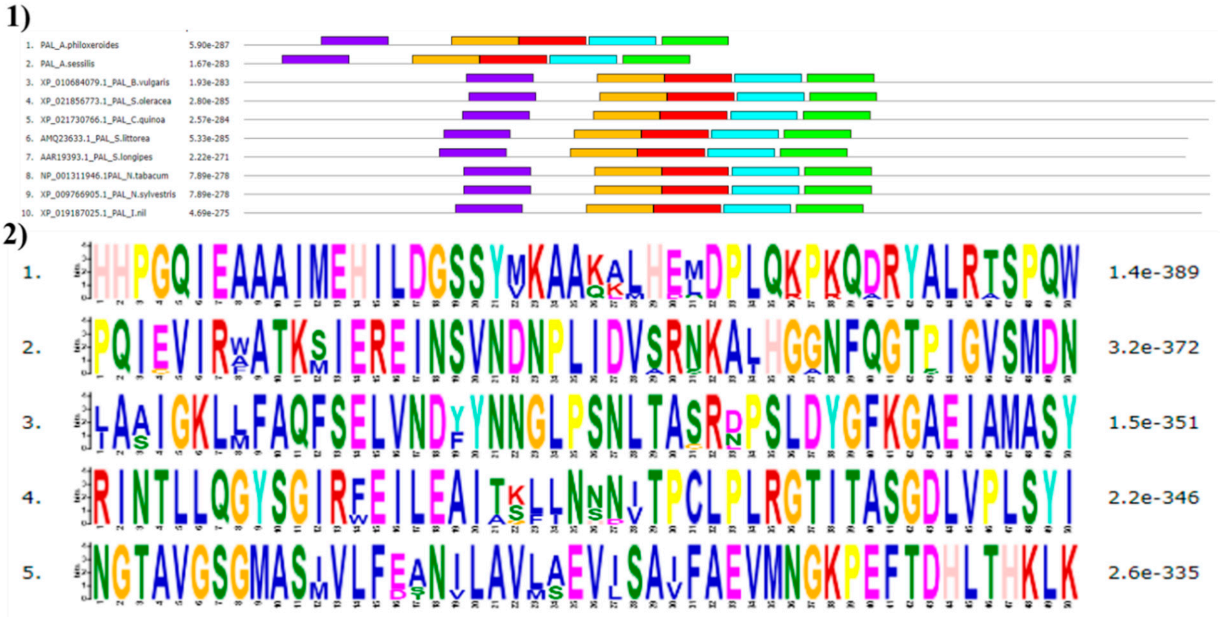

(b) *JAR1* (JA)

1)

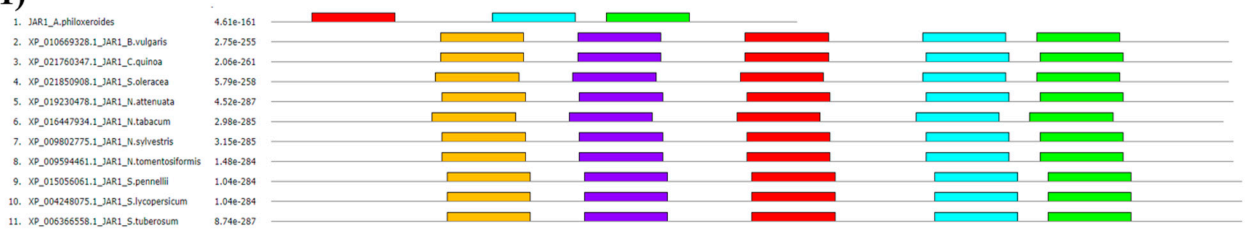

2)

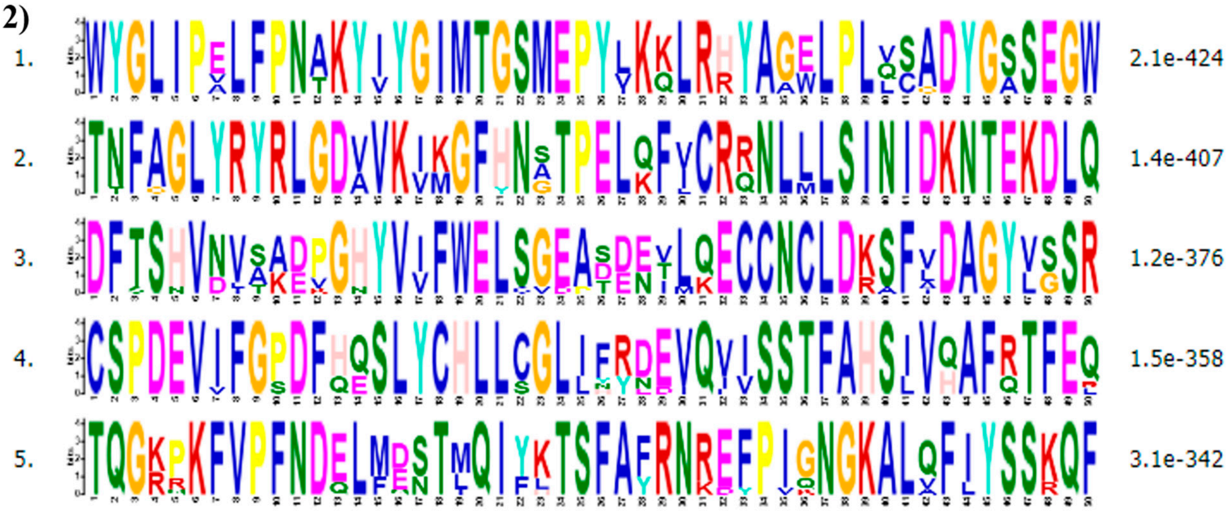

(c) *EIN3* (ET)

1)

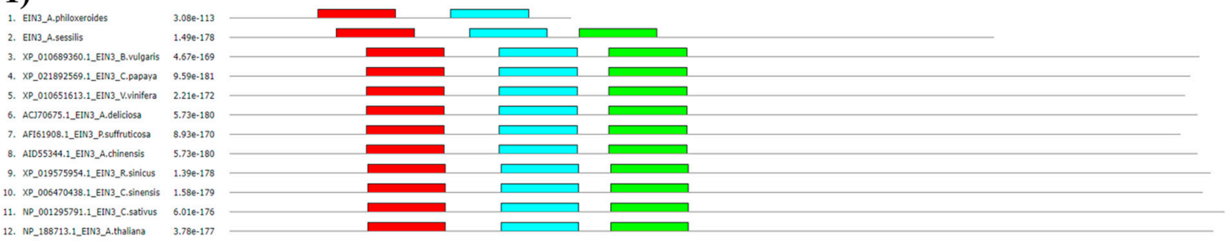

2)

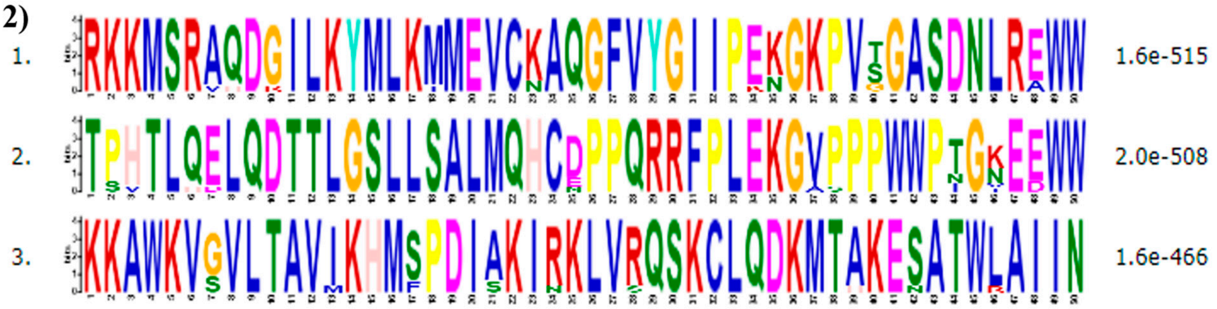

Figure S3. Identification of conserved motifs in selected genes from both invasive and native species. (a) *PAL* (Salicylic Acid), (b) *JAR1* (Jasmonic Acid), (c) *EIN3* (Ethylene). For each gene the following are shown: 1) a conserved motif revealing five motifs from the full-length protein sequences of all analyzed species with greater e-value, and 2) conserved amino acid sequences and the conservation of each motif are represented as a logo in each gene with their bit score as height of amino acid.

(a1) *PAL* (SA) - *A. philoxeroides*

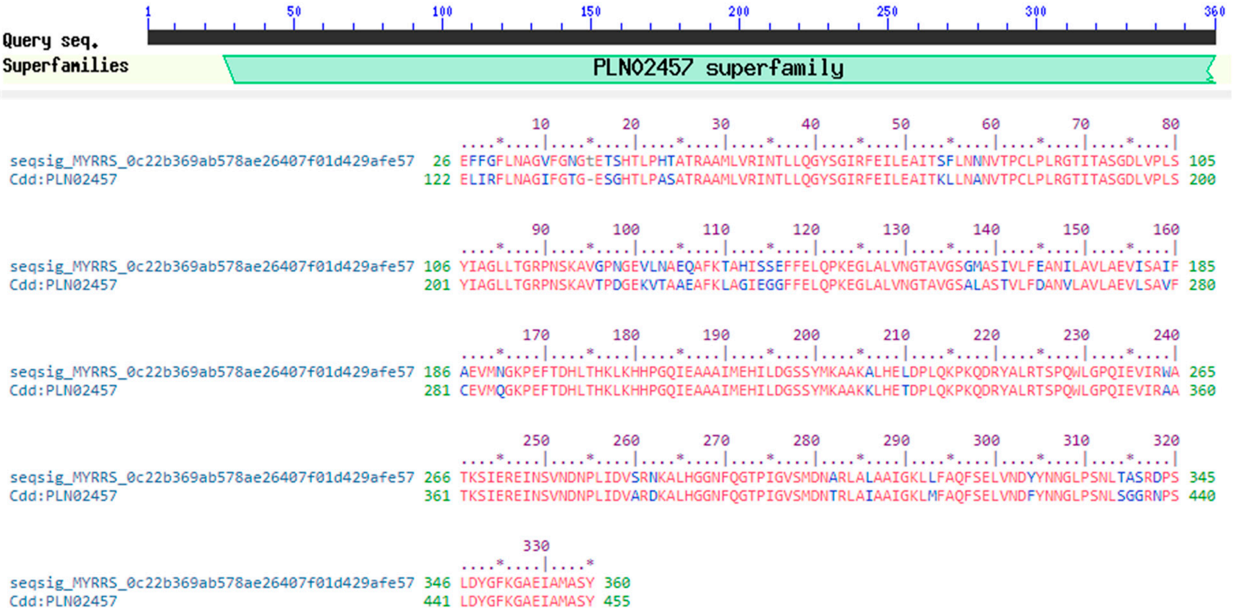

(a2) *PAL* (SA) - *A. sessilis*

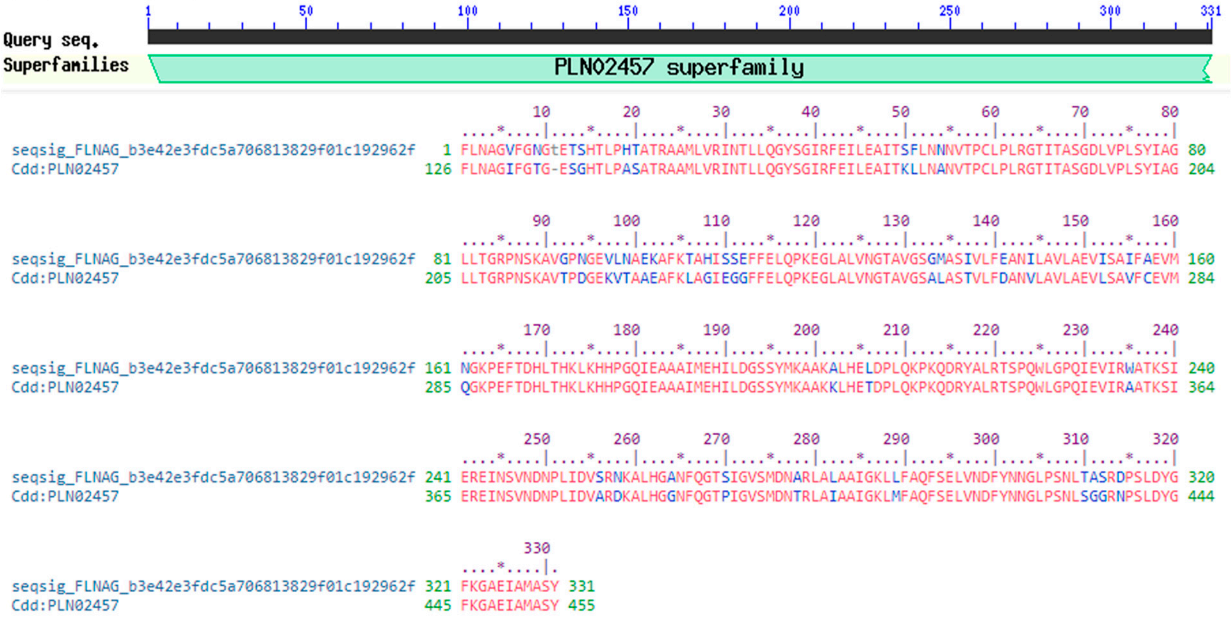

(b) *JAR1* (JA) - *A. philoxeroides*

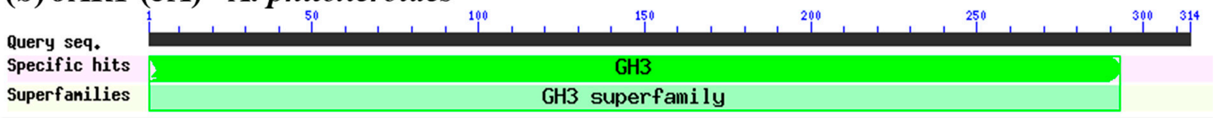


```
                                                        10        20        30        40        50        60        70        80
                                                ....*....|....*....|....*....|....*....|....*....|....*....|....*....|....*....|
seqsig_MSKLL_faa286a75f505e9cee82a46bf57179c0   1 MSKLLKPNPELATMIHRKCSSlsKWYGLIPELFPNAKYIYGIMTGSMEPYVKQLRRYAGWLPLVCGDYGSSEGWVAANVN 80
Cdd:pfam03321                                 238 LSKRLLPNPELADELERECEK--GWEGIIKRLWPNLKYVFCIGTGSMEQYIPKLRHYAGGLPLVSEGYGASEGFFGINLD 315

                                                        90       100       110       120       130       140       150       160
                                                ....*....|....*....|....*....|....*....|....*....|....*....|....*....|....*....|
seqsig_MSKLL_faa286a75f505e9cee82a46bf57179c0  81 PSAPPETATFAVLPNIGYFEFIPLNEsmNGEKESKTVGLHEVKVGQEYEIIVTTFAGLYRYRLGDAVKVMGFHNSTPELK 160
Cdd:pfam03321                                 316 PLCPPEDVSYTLLPNGAYFEFIPVDE--DEEDQPETLDLVEVEVGKEYELVVTTFSGLYRYRIGDVVRVTGFYNQTPRLE 393

                                                       170       180       190       200       210       220       230       240
                                                ....*....|....*....|....*....|....*....|....*....|....*....|....*....|....*....|
seqsig_MSKLL_faa286a75f505e9cee82a46bf57179c0 161 FVCRQNLLLSINIDKNTEKDLQVAVEEASKLLvaEKIDLIDFTSNVNVT-KEPGHYVIFWELSvEADENVMQECCNCLDK 239
Cdd:pfam03321                                 394 FVGRTNVVLSIFGEKTTEEDLQKAVEEAAKEL--TGAELVDYTSYADTSgSIPGHYVLFWELK-DDDPDVLEECCAELDE 470

                                                       250       260       270       280       290
                                                ....*....|....*....|....*....|....*....|....*....|....*.
seqsig_MSKLL_faa286a75f505e9cee82a46bf57179c0 240 SFVDA--GYVSSRKVGMIGPLELRVLRKGTFHKILLHSLSMGTTLNQFKTPRFVGS 293
Cdd:pfam03321                                 471 SLQEDnsDYRAKRKDGSIGPLEIRVVKPGTFDKLMDYAISRGASAGQYKVPRLVNS 526
```

(c1) *EIN3* (ET) - *A. philoxeroides*

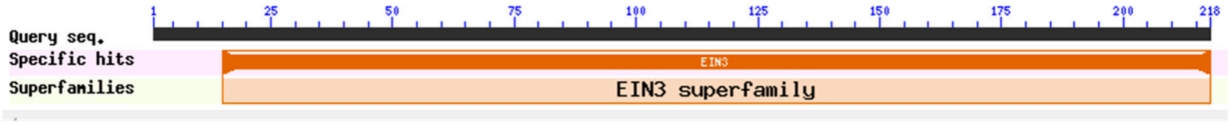


```
                                                       10        20        30        40        50        60        70        80
                                               ....*....|....*....|....*....|....*....|....*....|....*....|....*....|....*....|
seqsig_VGVEE_2094d2eb4b08d591fe2277b5426e61c6  15 DELEQRMWRDRMLLKKLKEQSKDK---AKEGVDAAKQRQSQEQARRKKMSRAQDRILKYMLKMMEVCKAQGFVYGIIPKN 91
Cdd:pfam04873                                   1 EDLEKRMWKDRMLLKRLKERQKGGlgaQLAALGKSKPKQPSEQARRKKMSRAQDGILKYMLKMMEVCNAQGFVYGIIPEK 80

                                                       90       100       110       120       130       140       150       160
                                               ....*....|....*....|....*....|....*....|....*....|....*....|....*....|....*....|
seqsig_VGVEE_2094d2eb4b08d591fe2277b5426e61c6  92 GKPVSGASDNLRAWWKEKVRFDRNGPAAISKYQADHSIPGTNEDCNavASTPHTLQELQDTTLGSLLSALMQHCDPPQRR 171
Cdd:pfam04873                                  81 GKPVSGSSDNLRAWWKEKVRFDRNGPAAIAKYQADNLLAGMSDESG--CSGPHSLHELQDTTLGSLLSALMQHCDPPQRR 158

                                                      170       180       190       200
                                               ....*....|....*....|....*....|....*....|....*..
seqsig_VGVEE_2094d2eb4b08d591fe2277b5426e61c6 172 FPLEKGAPPPWWPIGIEEWWPQLGLPKDQGPPPYKKPHDLKKAWKGS 218
Cdd:pfam04873                                 159 FPLEKGVPPPWWPTGKELWWGQLGLPKDQGPPPYRKPHDLKKAWKVS 205
```

(c2) *EIN3* (ET) - *A. sessilis*

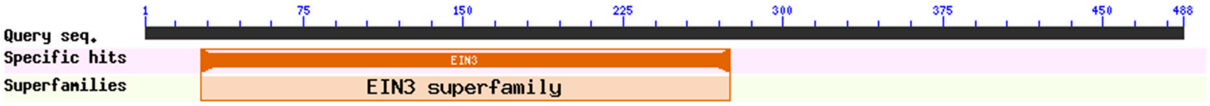


```
                                                       10        20        30        40        50        60        70        80
                                               ....*....|....*....|....*....|....*....|....*....|....*....|....*....|....*....|
seqsig_MGDEA_ae4829c9a4cc6f4c9a81d0141b7e1964  27 DELERRMWRDRMLLKKLKEQSKDK---AKEGVDAAKQRQSQEQARRKKMSRAQDGILKYMLKMMEVCKAQGFVYGIIPEK 103
Cdd:pfam04873                                   1 EDLEKRMWKDRMLLKRLKERQKGGlgaQLAALGKSKPKQPSEQARRKKMSRAQDGILKYMLKMMEVCNAQGFVYGIIPEK 80

                                                       90       100       110       120       130       140       150       160
                                               ....*....|....*....|....*....|....*....|....*....|....*....|....*....|....*....|
seqsig_MGDEA_ae4829c9a4cc6f4c9a81d0141b7e1964 104 GKPVSGASDNLRAWWKEKVRFDRNGPAAISKYQADHSIPGTNEDCNtvASTPHTLQELQDTTLGSLLSALMQHCDPPQRR 183
Cdd:pfam04873                                  81 GKPVSGSSDNLRAWWKEKVRFDRNGPAAIAKYQADNLLAGMSDESG--CSGPHSLHELQDTTLGSLLSALMQHCDPPQRR 158

                                                      170       180       190       200       210       220       230       240
                                               ....*....|....*....|....*....|....*....|....*....|....*....|....*....|....*....|
seqsig_MGDEA_ae4829c9a4cc6f4c9a81d0141b7e1964 184 FPLEKGVPPPWWPIGNEEWWPQLGLPKDQGPPPYKKPHDLKKAWKVSVLTAVIKHMSPDIAKIRKLVRQSKCLQDKMTAK 263
Cdd:pfam04873                                 159 FPLEKGVPPPWWPTGKELWWGQLGLPKDQGPPPYRKPHDLKKAWKVSVLTAVIKHMSPDISKIRKLVRQSKCLQDKMTAK 238

                                                      250
                                               ....*....|..
seqsig_MGDEA_ae4829c9a4cc6f4c9a81d0141b7e1964 264 ESATWLAIINQE 275
Cdd:pfam04873                                 239 ESDTWLAVLNQE 250
```

Figure S4. Identification and alignment of the conserved domain in each of the selected genes from both invasive and native species. We identified conserved domains for each gene: (a) *PAL* (Salicylic Acid), (b) *JAR1* (Jasmonic Acid), (c) *EIN3* (Ethylene). The upper figure represents the conserved domain, while the lower figure shows the respective domain peptide sequence aligned with related species using the NCBI domain database. We could not predict the conserved domain for *JAR1* due to the short number of amino acids from open reading frame.

(a) *PAL* (SA)

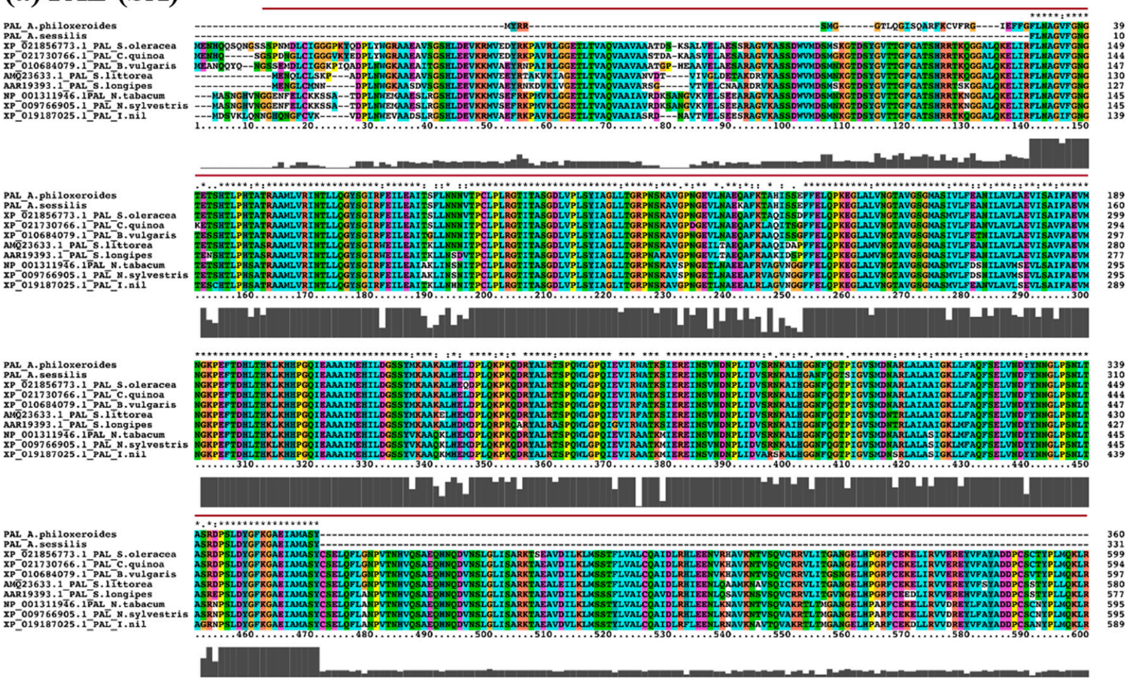

(b) *JAR1* (JA)

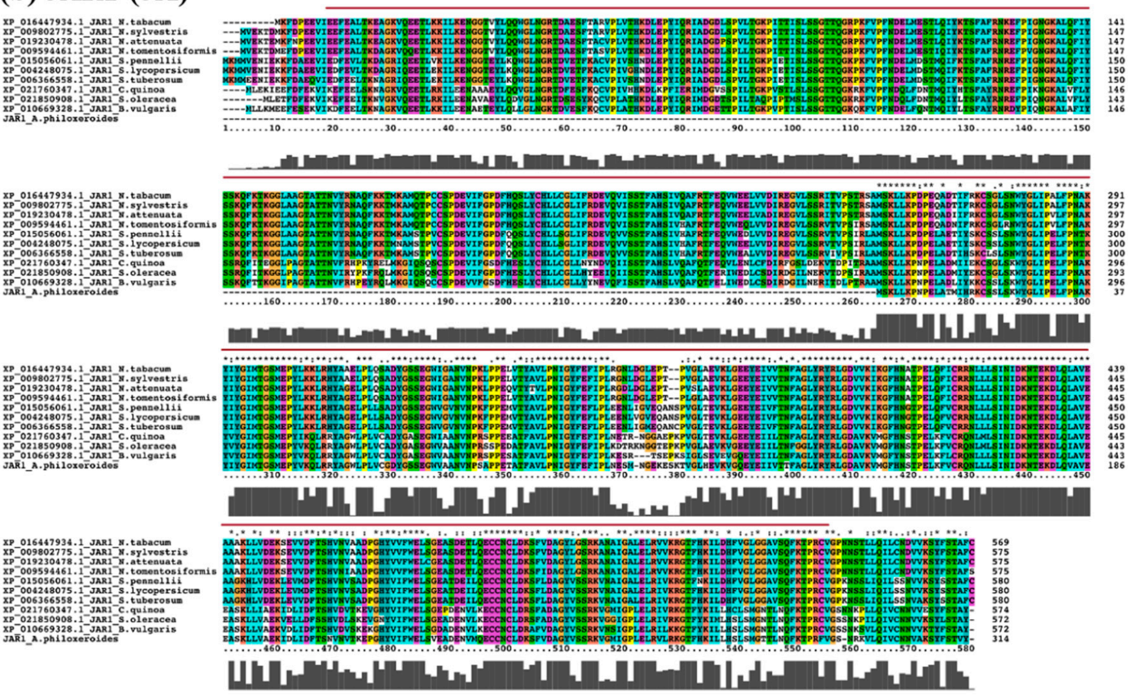

(c) *EIN3* (ET)

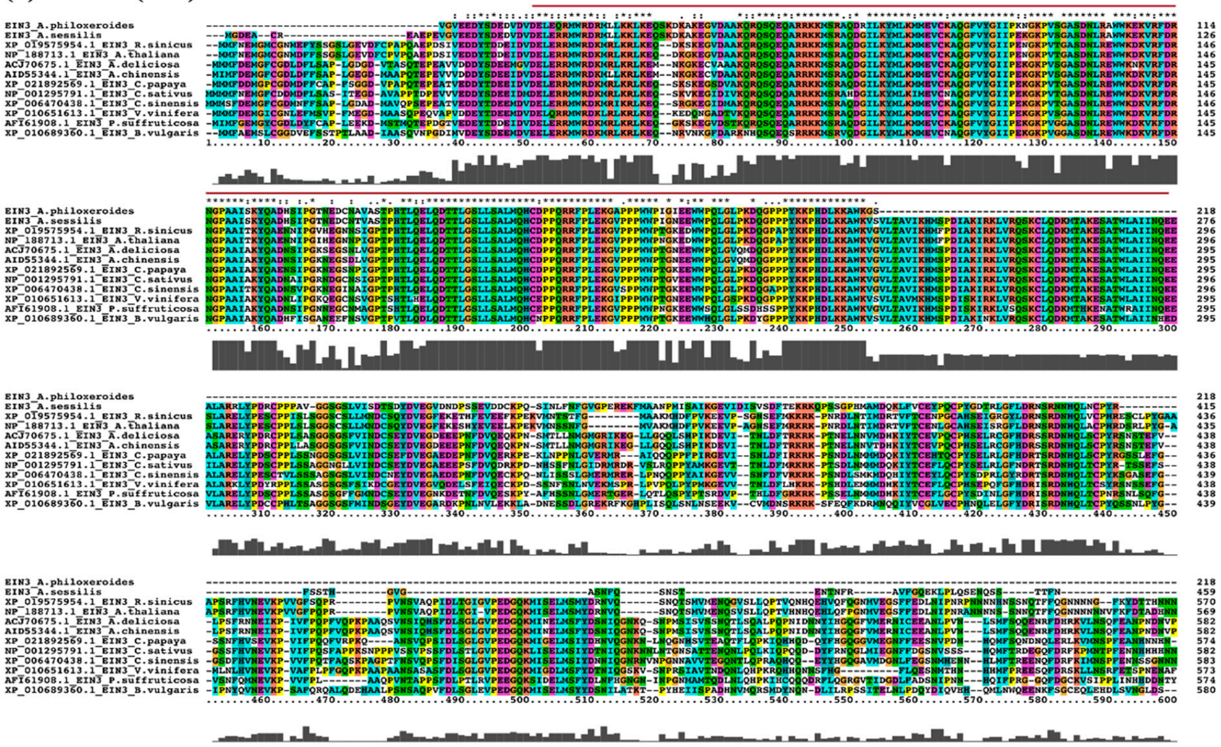

Figure S5. Multiple sequence alignments of predicted amino acid sequences of both *Alternanthera philoxeroides* and *A. sessilis* along with other closely related plants. (a) *PAL* (Salicylic Acid), (b) *JAR1* (Jasmonic Acid), (c) *EIN3* (Ethylene). The superfamily domain of each gene is highlighted with a red line. Star and colon symbols represent the conservation of amino acids and the dots and white space represent low conservation or mismatches respectively. Hyphens in the aligned sequences represent gaps in the sequence.

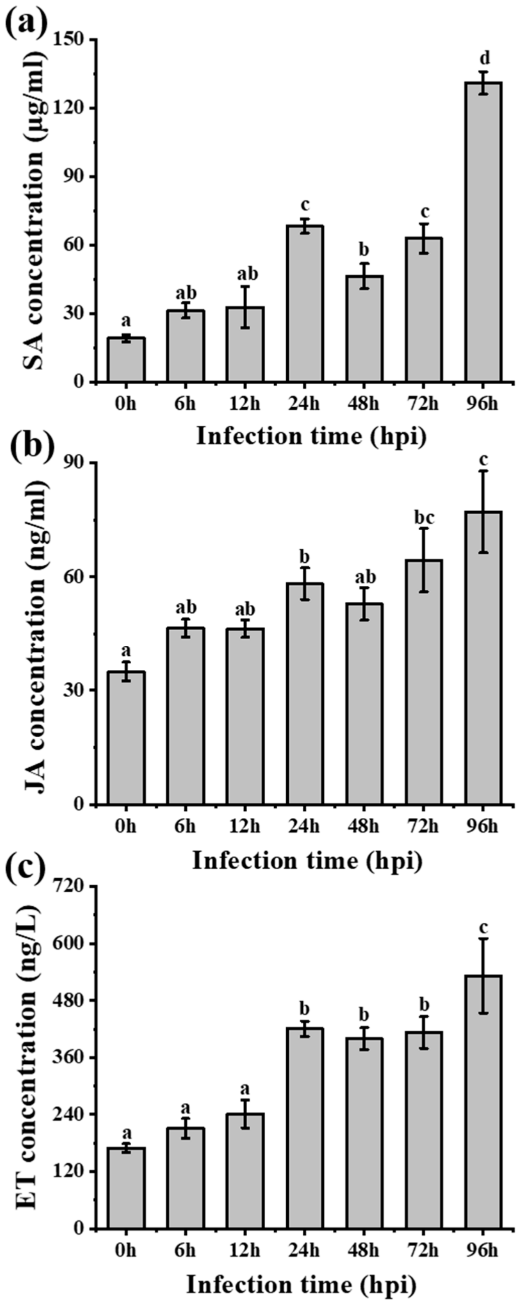

Figure S6. Endogenous hormone Salicylic acid (SA), jasmonic acid (JA), and ethylene (ET) contents in *Alternanthera philoxiroides* after infected with *Rhizoctonia solani*. (a) SA, (b) JA and (c) ET. Four-week-old plants were used for inoculations. Un-inoculated leaves were used as a control (0h). Samples were collected at different time intervals for quantification using ELISA. Values are means ± SE (n = 4). Data bars with different letters indicate they are significantly different compared with the un-inoculated leaves (using Duncan's test at $P < 0.05$).

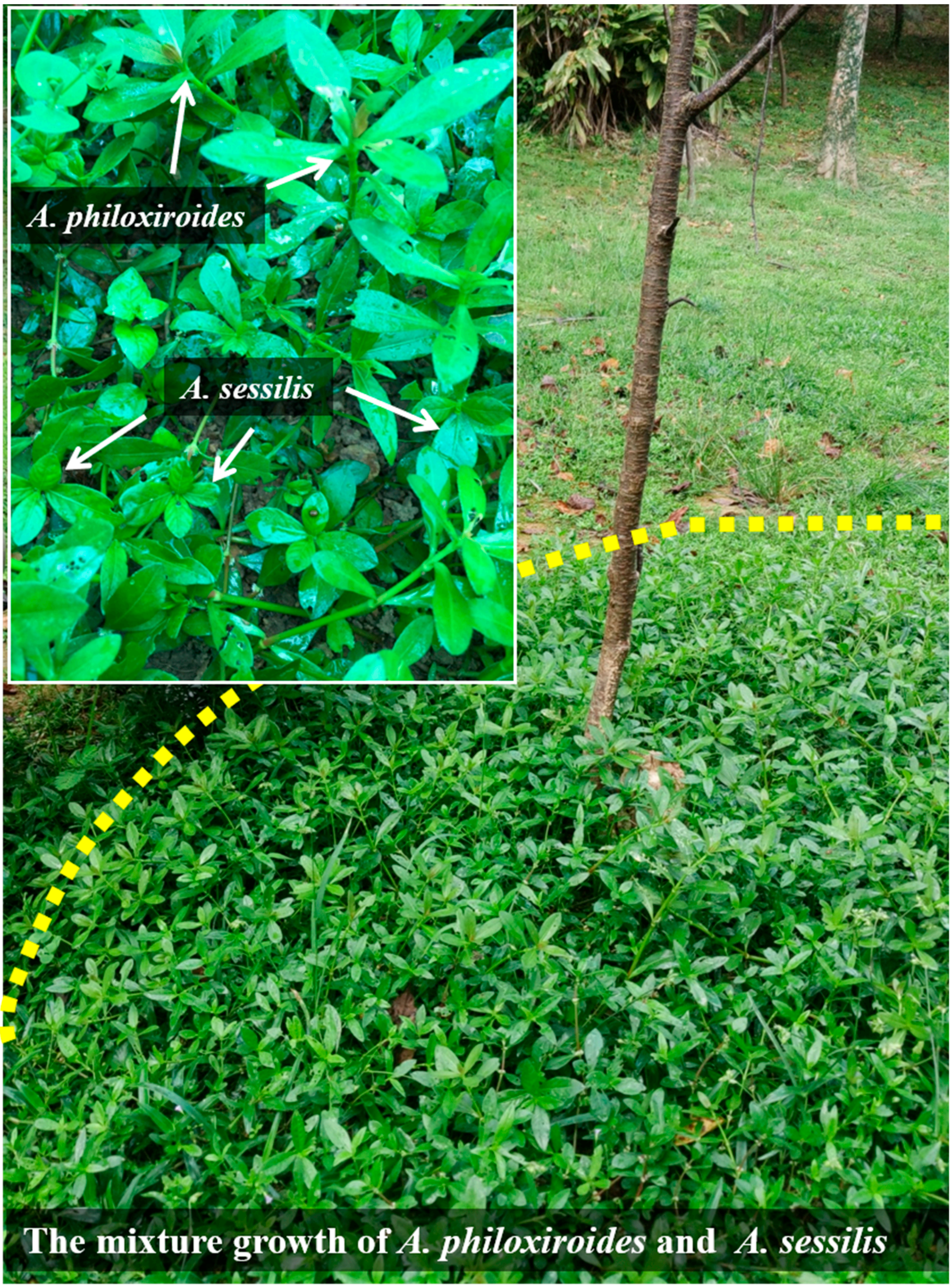

Figure S7. Sampling site of invasive *Alternanthera philoxeroides* and native *A. sessilis*. Samples were collected from the Fuzhou National Forest Park, Fuzhou, Fujian, China.

Table S1. Fold-change ratio of defense hormones and their responsive genes under different treatment conditions in invasive *Alternanthera philoxeroides* compared to native *A. sessilis*. Fold-change ratios are based on the relative amounts of signal molecules produced in *A. philoxiroides* and *A. sessilis* at the different time intervals. Fold-change ratios are calculated based on the comparative $2^{-\Delta\Delta CT}$ method normalized with the *Actin* gene at indicated time intervals after infection. Values highlighted in green show positive (higher) expression and red indicate negative (reduced) expression, compared with the control. Abbreviations: JA – Jasmonic acid, SA – Salicylic acid, ET – Ethylene, AP - *A. philoxeroides*, AS - *A. sessilis*. .

Treatment	Time (hpi)	JA-*LOX*		JA-*JAR1*		JA-*PR6*		SA-*PAL*		SA-*PR3*		ET-*EIN3*	
		**AP**	**AS**	**AP**	**AS**	**AP**	**AS**	**AP**	**AS**	**AP**	**AS**	**AP**	**AS**
Rhizoctonia solani	6	2.71	2.90	1.28	1.12	1.09	1.64	0.53	1.13	0.06	0.13	0.92	0.68
	12	0.43	1.09	0.60	0.92	0.69	0.83	1.30	2.21	0.04	0.16	1.25	0.79
	24	1.94	0.45	0.64	1.20	0.87	2.00	3.24	1.74	3.30	11.36	1.02	0.60
	48	0.79	1.89	0.47	1.59	0.60	2.32	8.81	3.02	10.24	10.62	1.67	0.98
	72	1.04	0.61	0.55	1.16	0.71	1.92	6.86	6.59	7.56	13.99	1.20	0.61
	96	1.75	0.32	0.62	0.66	0.97	0.93	4.96	7.58	7.72	4.84	2.66	0.83
MeJA pretreated before *R. solani* inoculations	6	0.27	4.15	0.32	1.90	0.27	0.80	0.21	5.11	4.01	1.91	0.49	9.88
	12	0.32	4.78	0.24	1.09	0.56	1.20	0.89	6.76	2.45	2.08	0.37	4.19
	24	0.40	1.88	0.64	1.97	0.57	1.32	0.37	3.21	6.95	10.54	0.66	1.93
	48	0.05	2.04	0.23	0.91	0.90	1.82	1.97	10.65	75.56	2.04	0.83	3.35
	72	0.10	1.44	0.07	0.67	0.39	1.04	3.23	8.73	73.16	1.41	0.62	2.46
	96	0.08	1.47	0.17	0.61	0.66	0.92	1.03	6.88	56.92	4.25	0.47	2.52
SA pretreated before *R. solani* inoculations	12	0.06	0.11	1.29	0.54	0.76	1.47	1.41	1.04	38.89	2.57	2.11	0.90
	24	1.70	0.80	1.18	0.73	2.13	1.36	1.62	1.70	56.04	1.20	1.53	1.46
	48	0.63	0.02	1.13	0.71	2.53	0.49	1.25	3.44	98.83	1.88	1.44	1.62
ET pretreated before *R. solani* inoculations	12	0.39	0.22	1.93	2.44	2.22	1.63	0.63	1.61	7.46	5.92	1.86	2.48
	24	2.01	1.39	1.78	1.91	1.41	2.88	0.87	1.50	1.33	2.31	1.01	2.71
	48	3.01	1.37	1.63	1.50	1.37	1.48	1.80	0.61	13.32	0.25	1.24	2.26
Systemic leaves	6	1.46	2.10	1.04	1.23	1.10	1.32	0.81	1.28	0.14	0.17	0.68	0.58
	12	0.78	1.11	0.60	0.68	0.80	0.49	0.83	1.46	0.02	0.04	0.76	0.37
	24	1.68	6.07	0.60	1.60	1.05	3.13	0.77	2.87	0.03	0.39	0.54	1.15
	48	1.56	3.37	0.86	1.49	1.33	1.65	1.33	1.28	0.18	0.24	0.58	1.06
	72	2.03	3.01	0.95	1.64	1.08	1.44	1.45	1.69	0.38	0.32	0.54	1.02
	96	1.81	3.80	0.74	1.85	1.03	1.86	1.31	1.46	0.05	0.21	0.47	0.70

Table S2. Primers used for quantitative RT-qPCR. *A species-specific primer was used for the *LOX* gene due to difficulty in finding common sequences between species. For other genes, a single common primer to both species was used for expression analysis.

Gene	*Source	Primers (5′ to 3′)	Product size (bp)	Reference
PAL	*A. philoxeroides & A. sessilis*	F: 5′-TCTATGGATAATGCAAGGTTGG-3′<br>R: 5′-GGTTTGAGGGAAGACCATTG-3′	106	This study
PR3	*A. philoxeroides & A. sessilis*	F: 5′-ATCTCATGGAACTACAACTACGG-3′<br>R: 5′-AGAAGCCTGTCTTGAAGGATATAA-3′	112	This study
LOX	*A. philoxeroides*	F: 5′-GGTCGCTGAAACAATCAATAAC-3′<br>R: 5′-TCTTGATAACAGCTGGGACATC-3′	86	This study
LOX	*A. sessilis*	F: 5′-GGTCGCTGAAACGATCAATAAC-3′<br>R: 5′-TCTTGATAACAGCTGGGACATC-3′	86	This study
JAR1	*A. philoxeroides & A. sessilis*	F: 5′-CTACTCGAGCTGCTATGTCAAA-3′<br>R: 5′-AGCCATACCATTTGCTTAAACTG-3′	96	This study
PR6	*A. philoxeroides & A. sessilis*	F: 5′-TGAAGCAAAGGTATGGGTGAA-3′<br>R: 5′-CACCAAGGTCTGAAGGAGTAATAG-3′	101	This study
EIN3	*A. philoxeroides & A. sessilis*	F: 5′-TGCTGAAGATGATGGAGGTTT-3′<br>R: 5′-CACCAGGCTCGAAGATTGT-3′	106	This study
β-Tubulin	*A. philoxeroides & A. sessilis*	F: 5′-CTACCTCTTTAGTGCTCATCTTACC-3′<br>R: 5′-CAAATGTGGGATGCCAAGAAC-3′	106	This study
Elongation factor 1-alpha	*A. philoxeroides & A. sessilis*	F: 5′-TTGCTTCTGACTCCAAGAATGA-3′<br>R: 5′-GCATAACCATTTCCAATCTGACC-3′	100	This study
Actin	*A. philoxeroides, A. sessilis* & *Beta vulgaris*	F: 5′-TCAATGTGCCTGCTATGTATGT-3′<br>R: 5′-GTGACTAACACCATCACCAGAG-3′	104	This study & Li & Smigocki, 2016

Table S3. List of putative defense hormones and responsive genes isolated in both *Alternanthera philoxeroides* and *A. sessilis*.

Plant	Hormone	Gene	Function	Size (bp)	GenBank Accession
A. philoxeroides	Salicylic Acid	*PAL*	SA biosynthesis	440	MK790145
A. sessilis				436	MK790146
A. philoxeroides	Salicylic Acid	*PR3*	Acidic chitinase	304	MK790153
A. sessilis				303	MK790154
A. philoxeroides	Jasmonic Acid	*LOX*	JA biosynthesis	578	MK790155
A. sessilis				613	MK790156
A. philoxeroides	Jasmonic Acid	*JAR1*	Catalyzes the synthesis of several JA-amido conjugates	305	MK790147
A. sessilis				307	MK790148
A. philoxeroides	Jasmonic Acid	*PR6*	Proteinase inhibitor	172	MK790149
A. sessilis				172	MK790150
A. philoxeroides	Ethylene	*EIN3*	A transcription factor and positive regulator of ET-responsive gene expression	525	MK790151
A. sessilis				527	MK790152

Table S4. Sequence information of six defense hormones and responsive genes isolated from *Alternanthera philoxeroides* and *A. sessilis*. See Supplementary Table S2 for additional information regarding each isolated gene. Selected ORF (Open Reading Frame) indicates six-reading frames of each isolated gene sequence. Abbreviations: AP - *A. philoxeroides*, AS - *A. sessilis*, CDS - Coding DNA Sequence.

Predictions	Salicylic Acid				Jasmonic Acid						Ethylene	
	PAL		*PR3*		*LOX*		*JAR1*		*PR6*		*EIN3*	
	AP	AS	AP	AS	AP	AS	AP	AS	AP	AS	AP	AS
Nucleotide (bp)	451	448	305	306	307	622	310	311	175	174	535	537
Selected ORF	+2	+3	+1	+3	+1	+2	-3	+1	-2	-1	+3	+2
CDS length (bp)	418	414	165	303	282	270	132	123	147	147	447	480
Peptide length (aa)	139	138	55	101	94	90	44	41	49	49	149	160
Similarity (Gene family)	Phenylalanine ammonia-lyase		EP3-like protein		Lipoxygenase 6 gene family		JAR1-like proteins		No significant similarity		Ethylene insensitive 3-like protein	

Table S5. Functional analysis of three hormone genes in both *Alternanthera philoxeroides* and *A. sessilis*. Assembled contigs and annotated gene sequences were used for predicting protein isoelectric point (http://bioinformatics.org/sms2/protein_iep.html) and protein molecular weight (http://bioinformatics.org/sms2/protein_mw.html). We could not predict the gene from the assembled contigs of *JAR1* from *A. sessilis*. Abbreviations: AP - *A. philoxeroides*, AS - *A. sessilis*.

**Predictions**	***PAL* (SA)**		***JAR1* (JA)**		***EIN3* (ET)**	
	**AP**	**AS**	**AP**	**AS**	**AP**	**AS**
Contig length (bp)	1411	1286	1284	1206	675	1698
Number of Exons	2	1	1	-	1	1
mRNA length (bp)	1080	993	945	-	654	1464
No. of amino acids	360	331	314	-	218	488
Isoelectric Point (pH)	7.37	6.68	8.04	-	9.34	6.34
Protein molecular weight (kDa)	39.01	35.67	35.29	-	24.98	54.95
No. of motifs	5	5	3	-	2	3
Representing domain	PLN02457 superfamily (PAL) (26 to 360 aa)	PLN02457 superfamily (PAL) (1 to 331 aa)	GH3 superfamily domain (1 to 293 aa)	-	EIN3 superfamily (15 to 218 aa)	EIN3 superfamily (27 to 275 aa)

Table S6. Screening and isolation of defense hormones and responsive gene sequences from both invasive *Alternanthera philoxeroides* and native *A. sessilis*. The highlighted green color in the table are the sequences of genes that were successfully isolated (see Supplementary Table S3 above).

S. No	Gene name and function	Primer Name	Primers (5′ to 3′)	Primer Length (bp)	Tm (°C)	GC (%)	Product size (bp)
	**Salicylic Acid regulated signaling pathway genes**						
1	*NDR1*<br>NDR1 (Non race-specific Disease Resistance 1); a plasma membrane protein, for CC-NBS-LRR subset of R genes.	AmNDR1-F<br>AmNDR1-R	F: 5′-TGCTAGAGGGGTAAAATGGGA-3′<br>R: 5′-ACCCAAAACACCCAACAACATAA-3′	21<br>23	58.15<br>59.22	47.62<br>39.13	250
2	*EDS1*<br>PAD4 (PhytoAlexin Deficient 4); lipase-like. Act upstream of SA in basal resistance to biotrophic pathogens & ETI initiated by TIR-NBS-LRR subset of R genes.	AmEDS1-F<br>AmEDS1-R	F: 5′-GTGGAATGAGGTAGCCCAGA-3′<br>R: 5′-CGATCCAGTGTCCTCGTTCT-3′	20<br>20	58.80<br>59.19	55<br>55	577
3	*NPR1*<br>NPR1 (Nonexpresser of PR genes 1); transcription cofactor. Positive regulator of SA and negative regulator of JA.	AmNPR1-F<br>AmNPR1-R	F: 5′-ACACAGAGCTTTGGAATCCG-3′<br>R: 5′-ATGCACAACCGATCCTTTGG-3′	20<br>20	58.19<br>58.82	50<br>50	375
4	*PR1*<br>Pathogenesis related protein 1	AmPR1-F<br>AmPR1-R	F: 5′-TCATCCTTGCATCAACCCCT-3′<br>R: 5′-AGCCTGTCGTCCCAAGTAAA-3′	20<br>20	59.00<br>58.95	50<br>50	328
5	*PAL* (see Table S2)<br>Phenylalanine Ammonia Lyase.	AmPAL-F<br>AmPAL-R	F: 5′-TCACCCAGGACAGATTGAGG-3′<br>R: 5′-GCTCGGAACAGTAAGAAGCC-3′	20<br>20	58.72<br>58.64	55<br>55	476
6	*PR2*<br>PR-2 (endo-1,3-beta-glucanase)	AmPR2-F<br>AmPR2-R	F: 5′-CGGCCATGCAAAATGTCCAA-3′<br>R: 5′-GCCGCATACACAGTGTCTAC-3′	20<br>20	60.04<br>58.72	50<br>55	331
7	*PR3* (see Table S2)<br>PR3-Acidic chitinase.	AmPR3-F<br>AmPR3-R	F: 5′-TCCATGCAACCCAAGTAAGC-3′<br>R: 5′-GGTTATTCCCTGGCGAAACG-3′	20<br>20	58.46<br>59.27	50<br>55	332
8	*PR5*<br>PR-5 (thaumatin-like)	AmPR5-F<br>AmPR5-R	F: 5′-CCATGGTTTTCAACGCTCCA-3′<br>R: 5′-GTTAAACCCGTCGACAAGGC-3′	20<br>20	59.04<br>59.49	50<br>55	212
	**Jasmonic Acid regulated signaling pathway genes**						
1	*JAR1* (see Table S2)<br>(JAsmonate Resistant 1); jasmonate-amino synthetase; a member of the GH3 family. JAR1 enzyme that produces the JA-Ile conjugate.	AmJAR1-F<br>AmJAR1-R	F: 5′-TGCAGTCCTGATGAAGTGGT-3′<br>R: 5′-GCATTCGGGAAAAGCTCAGG-3′	20<br>20	58.94<br>59.55	50<br>55	335
2	*COI1*<br>(COronatine Insensitive 1); ubiquitin-protein ligase, required for jasmonate-regulated defense.	AmCOI1-F<br>AmCOI1-R	F: 5′-AGGTTAAAGCGGCTCAGGAT-3′<br>R: 5′-GCCCGACATAACTGAGACCT-3′	20<br>20	59.09<br>59.18	50<br>55	352
3	*MYC2*<br>JIN1 (Jasmonate INsensitive 1); MYC2; TF & a positive regulator of JA-responsive genes such as *VSP2* and *LOX2*, whereas it acts as a negative regulator of JA/ET-responsive genes such as *PDF1.2*.	AmMYC2-F<br>AmMYC2-R	F: 5′-CGAGTTCGCCTTGTAATCGG-3′<br>R: 5′-ATATCTCCCCTGATTCCGGC-3′	20<br>20	59.08<br>58.72	55<br>55	513
4	*LOX* (see Table S2)	AmLOX-F	F: 5′-TTCCGCGTGATGAAGCTTTT-3′	20	58.77	45	470

	JA-mediated defense pathway marker gene.	AmLOX-R	R: 5′-AATGGCCTCTTCGACACTCA-3′	20	59.02	50	
5	*AOS*<br>Allene Oxide Synthase 1, chloroplastic.	AmAOS-F<br>AmAOS-R	F: 5′-GTTCTCTCCTACCTCGACCC-3′<br>R: 5′-TTCGTCACGTGAGATCCCAA-3′	20<br>20	58.61<br>59.04	60<br>55	471
6	*PR6* (see Table S2)<br>PR6 proteinase inhibitor.	AmPR6-F<br>AmPR6-R	F: 5′-CTGGAGTTTGCAAGGGTTGT-3′<br>R: 5′-AGCACCAAGGTCTGAAGGAG-3′	20<br>20	58.60<br>59.31	50<br>55	204
7	*PR9* Peroxidase	AmPR9-F<br>AmPR9-R	F: 5′-TTGTCAGGGGTTGTGATGGA-3′<br>R: 5′-AATTGCTTGAGAAACCGGCA-3′	20<br>20	58.86<br>58.67	50<br>45	302
	**Ethylene regulated signaling pathway genes**						
1	*ACC* synthase<br>Aminocyclopropane-1-carboxylic acid synthase is an enzyme that catalyses the synthesis of 1-Aminocyclopropane-1-carboxylic acid (ACC), a precursor for ethylene, from S-Adenosyl methionine.	AmACC-F<br>AmACC-R	F: 5′-AGAACAGGGGTGGAGCTTTT-3′<br>R: 5′-CCAAACACTGTGGCTCCATA-3′	20<br>20	59.15<br>57.80	50<br>50	254
2	*EIN3* (see Table S2)<br>EIN3 (Ethylene INsensitive 3); transcription factor. A positive regulator of ET-responsive gene expression.	AmEIN3-F<br>AmEIN3-R	F: 5′-GCGAGTGGTGGAAAGACAAG-3′<br>R: 5′-GGACACTCACTTTCCATGCC-3′	20<br>20	59.13<br>58.83	55<br>55	351
3	*ERF1*<br>AP2/ERF and B3 domain-containing transcription factor. A TF & positive regulator of JA and ET signaling.	AmERF1-F<br>AmERF1-R	F: 5′-AAATGGTCATTGGGGTGCAC-3′<br>R: 5′-GTCAGTGGCATAGTGGGCTT-3′	20<br>20	59.02<br>60.04	50<br>55	205
4	*ERF2*<br>Ethylene-responsive transcription factor ERF024. A TF & positive regulator of JA and ET signaling.	AmERF2-F<br>AmERF2-R	F: 5′-GGGAAGTGGGTCTCGGAAAT-3′<br>R: 5′-AGCTGCCTGAATGTCCCTT-3′	20<br>19	59.38<br>58.92	55<br>52.63	204
5	*PDF1.2-A*<br>Defensin-like protein AX1. ET/JA-mediated defense pathway marker gene.	AmPDF1.2A-F<br>AmPDF1.2A-R	F: 5′-TGAGATGAACATGGTGGTTGAG-3′<br>R: 5′-ACGGGCACACAGATTCCAAT-3′	22<br>20	58.32<br>59.96	45.45<br>50.00	150
6	*PDF1.2-B*<br>Defensin Ec-AMP-D2-like. ET/JA-mediated defense pathway marker gene.	AmPDF1.2B-F<br>AmPDF1.2B-R	F: 5′-CAACTGCAGAGGTAGGACCA-3′<br>R: 5′-TGTTTAGTGCAGAAGCATCGAC-3′	20<br>22	59.02<br>59.26	55.00<br>45.45	172
